# Expression conditions and characterization of a novelly constructed lipoprotein intended as a vaccine to prevent human *Haemophilus influenzae* infections

**DOI:** 10.1016/j.jbc.2023.105031

**Published:** 2023-07-16

**Authors:** Ravinder Kaur, Jill Mangiafesto, Karin Pryharski, Sailee Rasam, Robert Zagursky, Michael Pichichero

**Affiliations:** 1 Center for Infectious Diseases and Immunology, Rochester General Hospital Research Institute, Rochester, New York, USA; 2 Department of Biochemistry, State University of New York at Buffalo, New York, USA

**Keywords:** *Haemophilus influenzae*, OMP26, lipidated recombinant proteins, diacylation, triacylation

## Abstract

Bacterial lipoproteins are structurally divided into two groups, based on their lipid moieties: diacylated (present in Gram-positive bacteria) and triacylated (present in some Gram-positive and most Gram-negative bacteria). Diacylated and triacylated lipid moieties differ by a single amide-linked fatty acid chain. Lipoproteins induce host innate immune responses by the mammalian Toll-like receptor 2 (TLR2). In this study, we added a lipid moiety to recombinant OMP26, a native nonlipidated (NL) membrane protein of *Haemophilus influenzae*, and characterized it extensively under different expression conditions using flow cytometry, LC/MS, and MALDI-TOF. We also investigated the ability of NL and lipidated (L) OMP26 to induce *in vitro* stimulation of HEK Blue-hTLR2-TR1 and hTLR-TLR6 cells. Our L-OMP26 was predominantly expressed in diacylated form, so we employed an additional gene copy of apolipoprotein N-acetyltransferase enzyme (Lnt)-rich *Escherichia coli* strain that further acylates the diacyl lipoproteins to enhance the production of triacylated L-OMP26. The diacyl and triacyl versions of L-OMP26, intended as a vaccine for use in humans, were characterized and evaluated as protein vaccine components in a mouse model. We found that the diacyl and triacyl L-OMP26 protein formulations differed markedly in their immune-stimulatory activity, with diacylated L-OMP26 stimulating higher adaptive immune responses compared with triacylated L-OMP26 and both stimulating higher adaptive immune response compared to NL-OMP26. We also constructed and characterized an L-OMP26φNL-P6 fusion protein, where NL-P6 protein (a commonly studied *H. influenzae* vaccine candidate) was recombinantly fused to L-OMP26. We observed a similar pattern of lipidation (predominantly diacylated) in the L-OMP26φNL-P6 fusion protein.

Lipoproteins in bacteria are involved in a broad range of functions, including virulence, cell division, stress responses, cell architecture, outer membrane biogenesis, and peptidoglycan synthesis and remodeling (1, 2). These lipoproteins are covalently modified with a lipid moiety at the N-terminal conserved cysteine residue and usually are located on the extracellular surface of the cell membrane or on the periplasmic side of both the inner and outer cell membranes. In bacteria, the lipid-modified structures are typically in diacyl or triacyl form, and usually three conserved enzymes are involved in the biosynthesis of the triacyl form (3, 4), as illustrated in Figure 1. Apolipoprotein N-acyltransferase (Lnt) enzyme helps acylate diacyl lipoproteins on their cysteine α-amino groups, converting the structure into the triacyl lipoprotein form. The N-acylation step in Gram-negative *Escherichia coli* is required for sorting lipoproteins from the inner membrane to the outer membrane by the localization of lipoprotein (Lol) system (5, 6).

**Figure 1:**
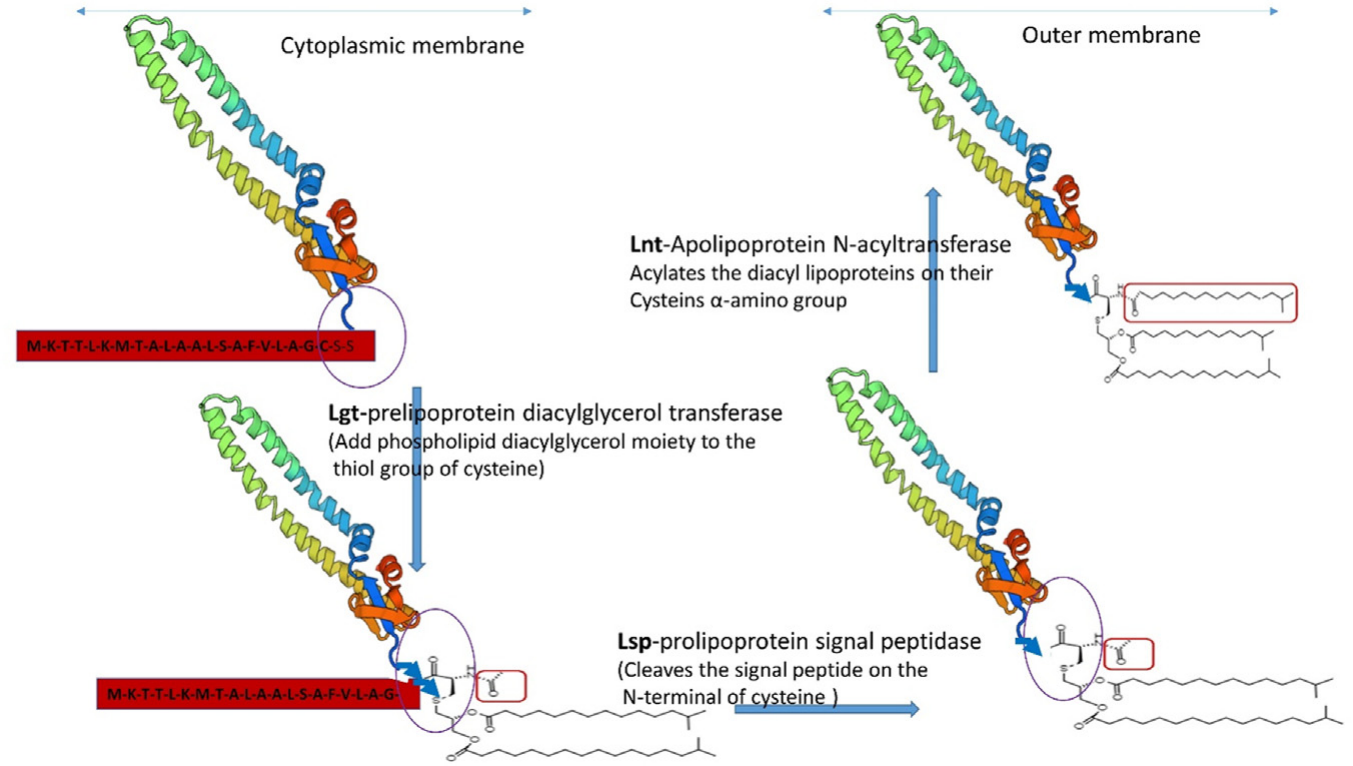
**Lipid modification pathway.** The expected pathway of lipid modification of OMP26 protein with different enzymes (Lgt, Lsp, and Lnt). The OMP26 homolog structure, Skp protein, is shown along with P4 protein signal sequence (*red* bar) that helps in lipid modifications. The structure of diacyl and triacyl modifications in lipoproteins is attached to the N-terminal cysteine residue of signal sequence of the protein.

Bacterial lipoproteins mainly exist in only one lipid-modified structure (4, 7). For example, *E. coli* contains only the triacyl lipoprotein form. However, recent work showed that lipoproteins in *Staphylococcus aureus* can exist in two lipidated forms, the diacyl and the triacyl form, modified by change in pH and bacterial growth phase (8). In addition, other environmental factors (*e.g.* salt concentration and temperature) have been shown to be involved in regulating the N-acyl state of *S. aureus* lipoproteins (8). The importance of diacyl- and triacyl-modified lipoproteins has been linked to invasiveness and commensalism, respectively, in *Staphylococci* (9). Lipoproteins are recognized by host pattern recognition receptors, such as Toll-like receptor-2 (TLR2), a heterodimer with TLR1 that recognizes triacylated lipoproteins or with TLR6 that recognizes diacylated lipoproteins. It has been generally held that regardless of which heterodimer is present, stimulation of TLR2/TLR1 or TLR2/TLR6 leads to inflammatory cytokine release and the establishment of adaptive immunity (10, 11, 12, 13, 14). The TLR1 ectodomain has a channel that binds the amide-bound lipid chain of the triacyl lipoprotein (15), while the same channel in TLR6 is obstructed by amino acid residues (16). TLR2 has a hydrophobic pocket that interacts with the remaining lipid chains in a less specific manner, allowing slight variations in length and chemical structure of the lipid. It is also thought that the same classical NFK-β signaling cascade is triggered, regardless of which of the two dimers is activated (13), although the kinetics may vary depending on the ligands, possibly resulting in different physiological outcomes (17, 18). Nguyen *et al* (9) showed that the TLR2 response induced by *S. aureus* and *Staphylococcus epidermidis* was almost ten times lower than that induced by *Staphylococcus carnosus*, which was partially due to their different modifications of their Lpp lipid moieties. They demonstrated that the triacyl N-acylated Lpp, recognized by TLR2-TLR1 receptors, silenced innate and adaptive immune responses, while the diacyl N-acetylated Lpp, recognized by TLR2-TLR6 receptors, boosted them (19). However, the mechanism by which these small lipid modifications dictate vaccination-induced immunity is not yet understood (9, 19).

It has been well established that lipidated proteins are highly immunogenic (20, 21, 22, 23, 24, 25). In a prior publication (26), we described the effects of lipidation (L) of recombinant proteins P6 and OMP26 compared to nonlipidated (NL) P6 and OMP26 in a mouse model. We reported that adding a lipid moiety to a recombinant protein that was not naturally lipidated, that is, OMP26, enhanced immunogenicity through TLR2 signaling of antigen-presenting cells and Th17 cell response in the nasal-associated lymphoid tissue. After intraperitoneal or intranasal vaccination, we showed that L-P6 and L-OMP26 induced approximately 10- to 100-fold-higher IgG antibody levels than NL-P6– and NL-OMP26–protected mice from nasopharyngeal colonization, a key step in infection pathogenesis, and middle ear infection. Also, fusion constructs of either L-P6 or L-OMP26 with the other protein in NL form significantly increased IgG antibody to both target proteins, even though only one of the proteins was lipidated. Nasal-associated lymphoid tissue cells from mice vaccinated with lipidated protein constructs had higher levels of interleukin-17 (IL-17), IL-22, and CD4+ T-cell memory.

Here, for the first time, we report preparation methods of L-OMP26 that preferentially enhance expression of the diacylated lipid form over the triacylated form. We describe characterization of the lipoprotein construct under different expression conditions using flow cytometry, LC-MS, and MALDI-TOF. We investigate the ability of NL- and L-OMP26 to induce *in vitro* stimulation of HEK Blue-hTLR2-TR1 and hTLR-TLR6 cells after differing conditions of protein expression, and we describe the downstream effects of expressing our protein in an apolipoprotein N-acetyltransferase enzyme (Lnt)-rich *E. coli* strain, which further acylates the diacyl lipoproteins to enhance the production of triacylated L-OMP26. We also characterized and evaluated the diacyl and triacyl versions of L-OMP26 as protein vaccine components to prevent *Haemophilus influenzae* infections in a mouse model to assess their immune-stimulatory activity. Finally, we constructed an L-OMP26φNL-P6 gene fusion, where NL-P6 protein (a commonly studied *H. influenzae* vaccine candidate) was genetically fused to L-OMP26 and characterized the recombinantly expressed fused protein to determine if a similar pattern of lipidation (diacylated predominant) in L-OMP26φNL-P6 fusion persisted and produced the same effects on immunity as L-OMP26 alone.

## Results

Expression of L-OMP26 in BL21 (DE3) *E. coli* cells did not result in an appreciable yield of the target protein. Therefore, we transformed the L-OMP26 gene construct into C43 (DE3) *E. coli* to enable the expression of the lipidated protein (27). The recombinant L-OMP26 proteins expressed in low yield in nutrient rich (2X-YT) media, so we switched to a nutrient-deficient minimal media, M9-selected (M9-MM) (28) to increase the yield of the target lipidated proteins and to ensure development into the mature form (without signal sequence) of lipidated proteins. The Coomassie blue–stained SDS-PAGE gel shows expression levels of L-OMP26 and the L-OMP26φNL-P6 fusion protein under different growth conditions (Fig. S1). We were not able to monitor immature (still with signal sequence) and mature forms of the lipoprotein (diacyl and triacyl) under different conditions. We recovered a final yield of 1-2 mg/liter of lipidated protein after purification. The amount of endotoxin in each protein formulation was negligible. The amount given to mice is shown in Table S1.

### Comparison of surface expression of L-OMP26 on *E. coli* in two different media conditions and growth phase

**Figure 2:**
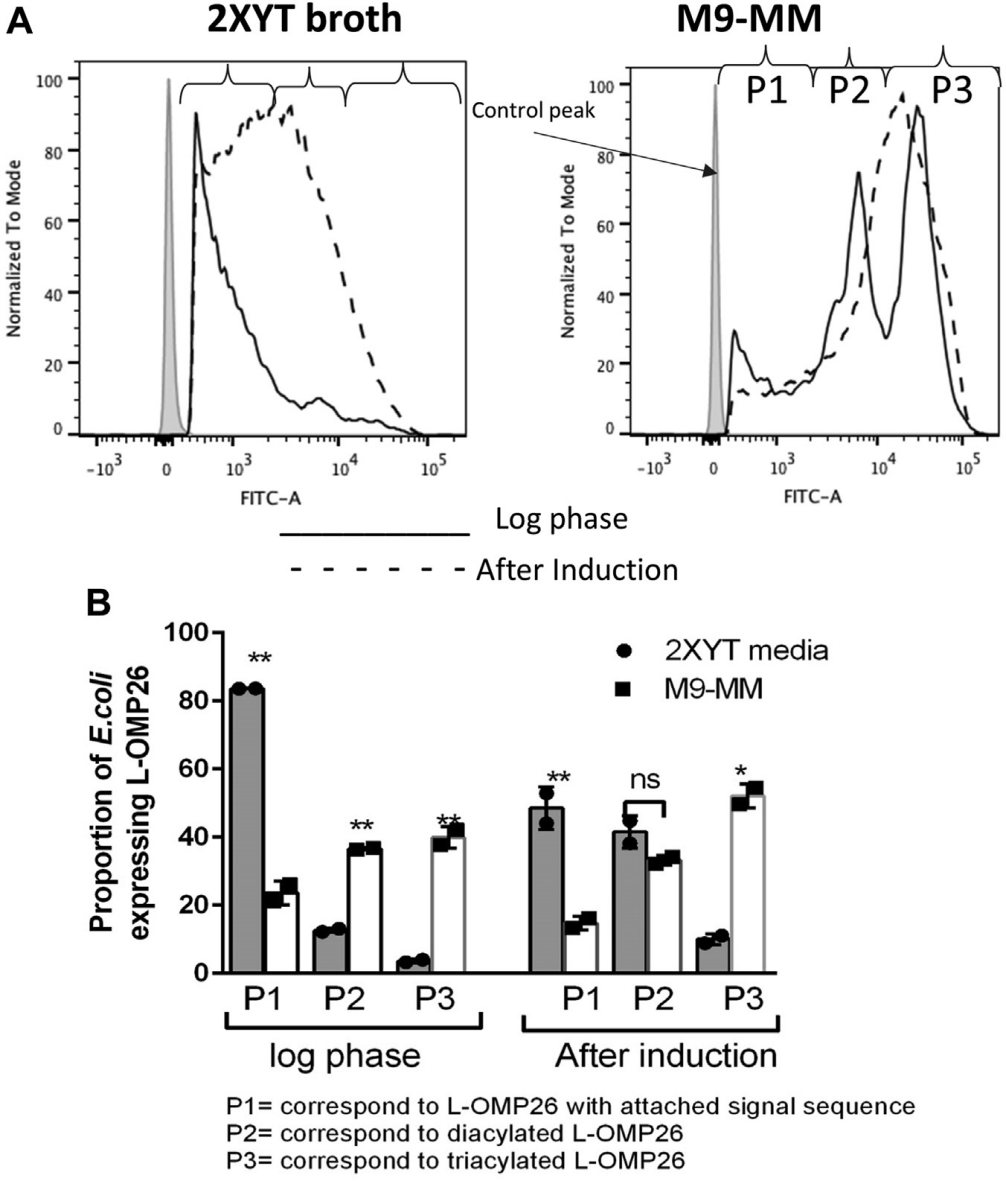
**Surface expression of L-OMP26 in different media.***A*, *Escherichia coli* surface expression determined by flow cytometry of L-OMP26 grown in 2X-YT media and M9-MM grown to log phase (*solid line*) and after induction with IPTG (*dotted line*). Three peaks (P1, P2, and P3) were assigned at log phase. Based on the known biology of lipoprotein transport to bacterial membranes, P1 corresponds to diacylated L-OMP26 with attached signal sequence, P2 corresponds to diacylated L-OMP26 without signal sequence, and P3 corresponds to triacylated L-OMP26 with no signal sequence. *B*, population density area of P1, P2, and P3 peaks shown as proportion expression corresponding to 2X-YT media and M9-MM (mean±SD are plotted). *p*-values determined by *t* test. ∗*p* < 0.05; ∗∗*p* < 0.01; ns=not significant. M9-MM, minimal media, M9-selected.

Figure 2 shows the *E. coli* surface expression levels of L-OMP26 grown in 2X-YT media and M9-MM, as determined by flow cytometry. For triacylated proteins, after the protein is lipidated, the signal sequence is cleaved, then the lipidated protein is transported by the lol pathway system to the outer membrane of the *E. coli* and becomes surface exposed (Fig. 1), where epitopes are available to bind to FITC-labeled antibodies directed to the protein. The lipoprotein goes to the surface of the *E. coli* only if it is diacylated or triacylated (as shown in Fig. 1). The higher the amount of lipoprotein that is surface exposed on the *E. coli*, the more it can bind to the FITC-labeled antibody and a greater shift in fluorescence will be detected by flow cytometry. For diacylated proteins, after the protein is lipidated, the lipidation signal sequence may be retained or cleaved. If the signal sequence is retained, then the product will translocate to the outer membrane of *E. coli* with poor efficiency and the protein will not be fully surface exposed resulting in lower FITC-antibody labeling. If the signal sequence is cleaved, then the product will be transported fully to the outer membrane resulting in increased FITC-antibody labeling (Fig. 1). Diacylated lipoproteins without signal sequence will have less surface expression than triacylated lipoproteins*.* Based on the known biology of lipoprotein transport to bacterial membranes shown in Figure 1, the three major peaks in the flow spectra of the experiments would correspond with three versions of the OMP26 protein: diacylated L-OMP26 with signal sequence (peak 1), diacylated L-OMP26 without signal sequence (peak 2), and triacylated L-OMP26 with no signal sequence (peak 3). The population density area of each of these three peaks was plotted to quantitate the percentage of surface expression when the bacteria was cultured in either 2X-YT media or M9-MM (Fig. 2*B*). The *E. coli* pet21a T7 transcription system is leaky, which is why we observed expression of our L-OMP26 protein prior to induction with IPTG. In log phase, we observed a significant increase in the P2 and P3 peak areas when *E. coli* was grown in M9-MM (*p* < 0.01) compared to 2X-YT media. The expression of P1 was higher (*p* < 0.001) in 2X-YT media indicating that L-OMP26 does not modify into its mature diacylated and triacylated forms (with signal sequence cleavage) in nutrient-rich media. The different media types did not yield a significant difference in the P2 population, post-IPTG induction, but we did observe an increase in the P3 peak (*p* < 0.05) and a decrease in the P1 peak (*p* < 0.01) in M9-MM compared to 2X-YT media. As a control, NL-OMP26 was also expressed in *E. coli*. Flow cytometry experiments on this sample showed no surface labeling, as expected for a nonsurface-exposed protein (data not shown).

### Mass spectrometry comparison of acylation modifications to purified L-OMP26 from *E. coli* cultured under two different media conditions

The MS/MS spectrum of the ion at m/z 792.928 provided y- and y∗-series ions, describing modification B (C16:0, C16:1) of the diacylated peptide sequence of L-OMP26 (Fig. S2). An example of triacyl peptide modification 1 at m/z 852.487 is shown, representing C16:0, C16:1, C16:0. Modification 1 was compared between L-OMP26 spectra from *E. coli* grown in 2X-YT media and M9-MM. No triacyl modification was observed (we considered a peak intensity <1000 to be base noise) for L-OMP26 from *E. coli* grown in 2X-YT media. Similarly, possible lipid modifications (1, 2, 3, A, A′, B, B′, C, D, E corresponding to different lengths and types of lipid chains) were compared (Table 1) for L-OMP26 from *E. coli* grown in 2X-YT media and M9-MM. L-OMP26 from 2X-YT media did not show appreciable lipid modifications (only diacyl peaks 1 and 2 were observed), suggesting a lack of mature lipidated protein. We observed a high intensity peak representing the L-OMP26 peptide with signal sequence of L-OMP26 grown in 2X-YT media, but not in M9-MM (data not shown), again suggesting a lack of maturation. Table 1 also shows the ratio of diacyl/triacyl peak intensities for L-OMP26 expressed in *E. coli* grown in M9-MM, indicating that L-OMP26 was predominantly diacylated when expressed in *E. coli* grown in M9-MM.

**Table 1:** Mass spectrometry analysis of lipidation status of L-OMP26 from *Escherichia coli* grown in 2XYT and M9-MM media showing peak areas of different lipid modifications (triacyl and diacyl peaks) including the ratio of diacyl *versus* triacyl

Lipid modification!		L-OMP26 express in 2X-YT			L-OMP26 express in M9-MM		
		Peak areas observed by mass spectrometry					
		Triacyl peak area	Diacyl peak area	Ratio of diacyl *versus* triacyl	Triacyl peak area	Diacyl peak area	Ratio of diacyl *versus* triacyl
1	C16:0, C16:1	-	4.3 E+01	-	1.74 E+06	2.55 E+07	14.67
2	C16:0, C17:cyclo	-	1.7 E+04	-	1.67 E+07	4.15 E+08	24.87
3	C16:0, C18:1	-	-	-	1.69 E+06	6.25 E+07	36.94
A	C14:0, C16:1	-	-	-	2.84 E+04	6.18 E+05	21.73
A'	C16:1, C16:1	-	-	-	-	1.15 E+05	-
B	C15:0, C16:1	-	-	-	2.39 E+05	2.85 E+06	11.89
B'	C14:0, C16:0	-	-	-	1.34 E+06	2.41 E+07	17.98
C	C16:1, C18:1	-	-	-	-	5.38 E+05	-
D	C18:1, C18:1	-	-	-	-	8.19 E+06	-
E	C16:0, C19:cyclo	-	-	-	-	1.84 E+06	-
	Sum of all peaks	-	1.7 E+04	-	2.17 E+07	5.41 E+08	24.91
! In triacyl, C16:0 chain was added in theoretical calculations							

Note: Intensity of <5 × 10^3^ was not considered a significant peak but considered noise(−).

We also acquired MALDI-TOF spectra of L-OMP26 expressed in *E. coli* cultured in M9-MM with and without additional Lnt enzyme co-expression. The spectral differences are shown in Fig. S3. Overall, we observed the same MS signature for L-OMP26 in both spectra, and any differences were related to the lipidation moiety changes (see inset magnified view). Highlighted ions at m/z that were different in two spectra are further magnified in Fig. S3. The position of O-acylation was not determined.

### Impact of pH on the surface expression of L-OMP26 on *E. coli*

**Figure 3:**
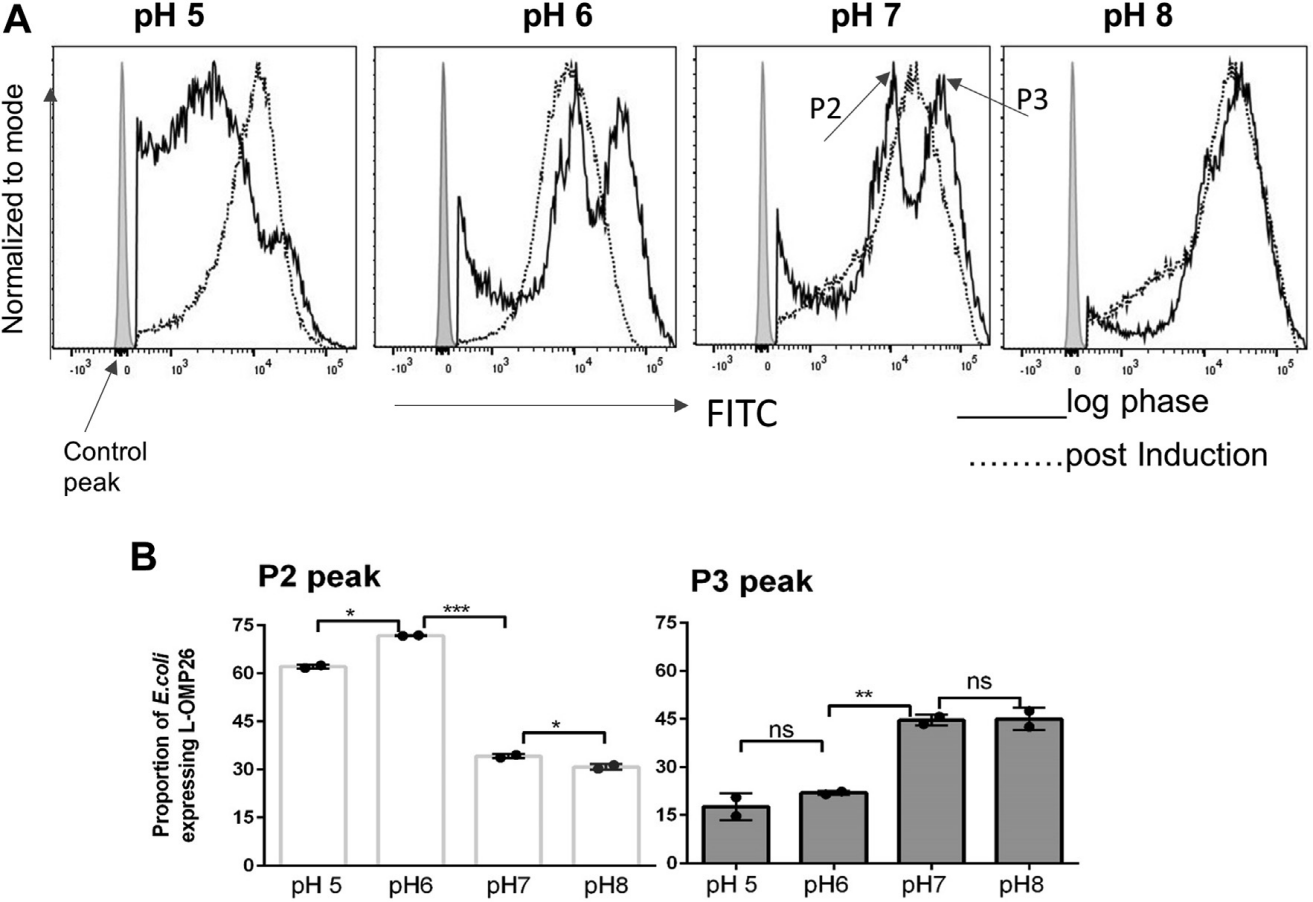
**Surface expression of L-OMP26 with change in pH.***A*, change in expression of L-OMP26 on the surface of *Escherichia coli* with change in pH at log phase (*solid line*) and postinduction phase (*dotted line*). Two peaks (P2 and P3) were assigned at log phase. P2 corresponds to diacylated L-OMP26 and P3 corresponds to triacylated L-OMP26. *B*, population density area of P2 and P3 peaks shown (mean ± SD) as proportion expression corresponding to each pH. *p*-values determined by *t* test between two groups. ∗*p* < 0.05; ∗∗*p* < 0.01; ∗∗∗*p* < 0.001, ns = not significant.

Lnt enzyme activity is sensitive to pH (29). At pH above 7, Lnt enzyme activity increases, which would expect to increase triacylation of lipoproteins. To study the effect of varying pH on acylation state, we examined whether different pH conditions affected the surface expression of diacyl or triacyl L-OMP26 on *E. coli* cultured in M9-MM buffered at pH 5, 6, 7, and 8 (Fig. 3), based on the rationale that environmental conditions (like pH, temperature, salt) affect the degree of fatty acid acylation (30). At pH 5 and 6, *E. coli* cells produced more diacylated L-OMP26, represented by P2 peaks, postinduction. The maximum P2 peak intensity was at pH 6 and only slightly lower (*p* < 0.05) at pH 5, due to slower *E. coli* cell growth at pH 5. The percentage of diacylated L-OMP26 at pH 6.0 was higher than the percentage at pH 7 (*p* < 0.01) and pH 8 (*p* < 0.05). In contrast, M9-MM buffered at pH 7 and pH 8 resulted in higher amounts of (*p* < 0.01) triacylated L-OMP26 (P3), postinduction.

### Comparison of reactogenicity and immunogenicity after vaccination with L-OMP26 from *E. coli* grown in two different media conditions

**Figure 4:**
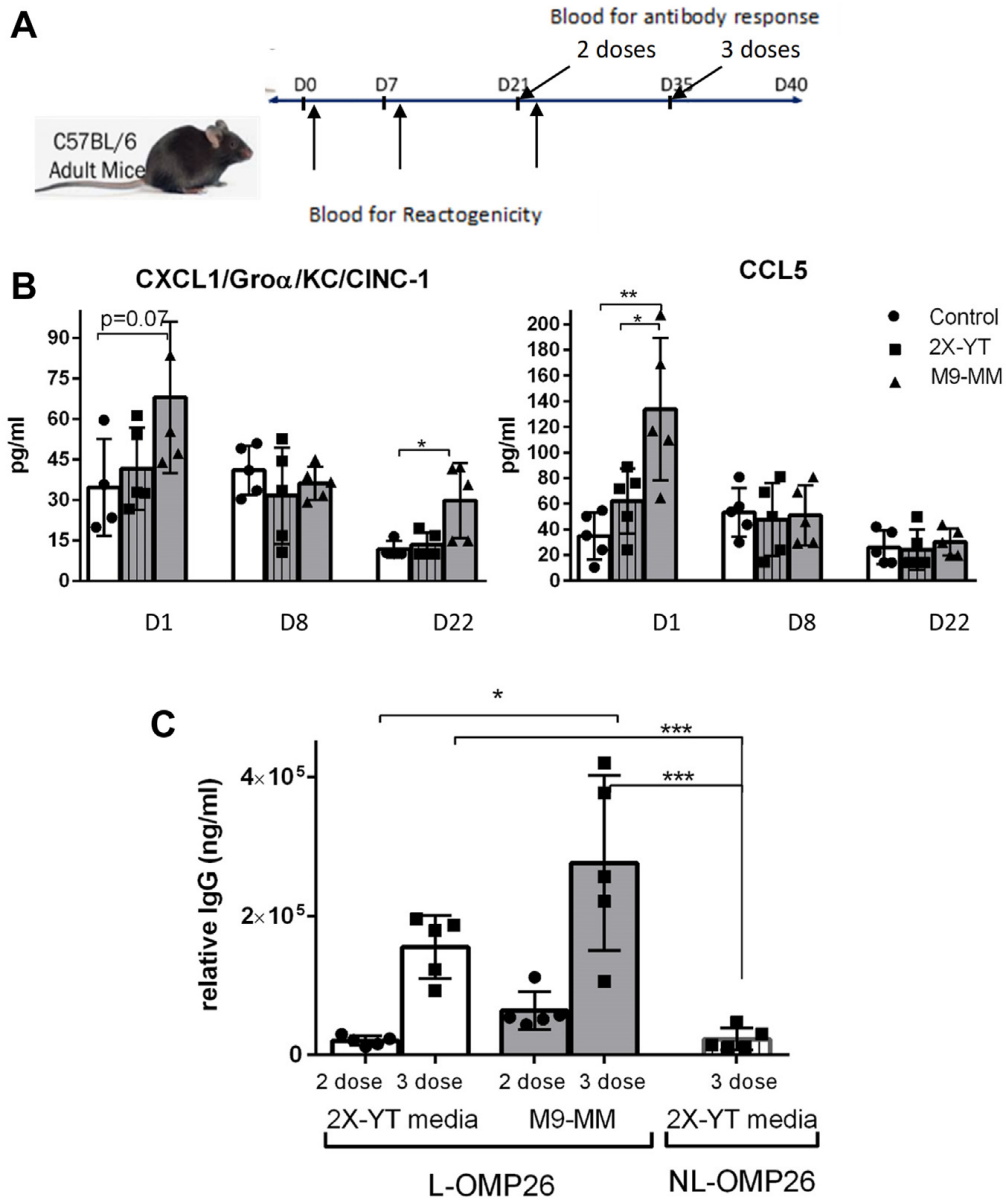
**Reactogenicity and immunogenicity in mice with L-OMP26 vaccination.***A*, vaccination schedule in mice for reactogenicity and immunogenicity. Sera was collected 24 h after each L-OMP26 immunization (5 μg dose/mice). *B*, comparison of CXCL1/Groα/KC/CINC-1 (equivalent to IL-8) and CCL5 in mice immunized with different constructs of L-OMP26. *p*-values by ANOVA with Tukey multiple comparison. *C*, comparison of IgG levels at D22 and D35 for L-OMP26 after *Escherichia coli* were grown in 2X-YT media and M9 minimal media (M9-MM). Antibody levels induced by NL-OMP26 after *E. coli* were grown in 2X-YT media were included for comparison with L-OMP26. *p*-value by ANOVA with multiple comparisons after three doses of L-OMP26 or NL-OMP26. ∗*p* < 0.05; ∗∗*p* < 0.01; ∗∗∗*p* < 0.001 and ns = not significant. IL, interleukin; NL, nonlipidated.

Blood samples for reactogenicity and/or immunogenicity were taken after 24 h postvaccination at three time points (days 0, 7, and 21; Fig. 4*A*). Based on the premise that more lipid in the vaccine construct would result in higher reactogenicity, we measured inflammatory cytokine/chemokine levels in mice after vaccination with L-OMP26 grown in M9-MM at pH 7 (presumably with higher levels of mature L-OMP26 and greater diacylated L-OMP26 expression) and 2X-YT media (presumably with lower levels of mature L-OMP26 and lower diacylated L-OMP26 expression). Pro-inflammatory cytokine CXCL1/Groa/KC/Cinc-1 (IL-8 equivalent that is a chemoattractant for neutrophils) levels and chemokine CCL5 (recruits T cells, monocytes, and dendritic cells) levels were higher (*p* < 0.01 for both) 24 h after the first vaccination dose with L-OMP26 from *E. coli* grown in M9-MM compared to L-OMP26 from *E. coli* grown in 2X-YT media (Fig. 4*B*). No difference was observed after the second vaccination, but again after the third vaccination, IL-8 (*p* < 0.05) and CCL5 (*p* < 0.05) levels were higher with L-OMP26 purified from *E. coli* grown in M9-MM compared to 2X-YT media (Fig. 4*B*). Thus, L-OMP26 from *E. coli* grown in M9-MM (presumably with higher lipid content) was associated with a higher pro-inflammatory cytokine response overall. Cytokines/chemokines CCL2/MCP-1, IFN-γ, IL-6, IL-17/IL-17A, CXCL2GRO β/MIP-2/CINC-3, IL-1β, IL-10, and TNF-α were undetectable after all three vaccinations.

For immunogenicity, both L-OMP26 formulations (L-OMP26 expressed in *E. coli* grown in 2X-YT media and M9-MM) elicited higher antibody IgG levels at day 22 and day 35 after vaccination (*p* < 0.001, both time points) compared to NL-OMP26 (∼10 fold higher; Fig. 4*C*). L-OMP26 from *E. coli* grown in M9-MM (presumably with higher diacyl lipid content) had higher IgG levels at day 22 and 35 time points (around two fold higher; *p* < 0.05) than L-OMP26 from *E. coli* grown in 2X-YT media.

### Impact of additional Lnt enzyme on the surface expression of L-OMP26 on *E. coli* and on acylation modifications and immunogenicity of L-OMP26

**Figure 5:**
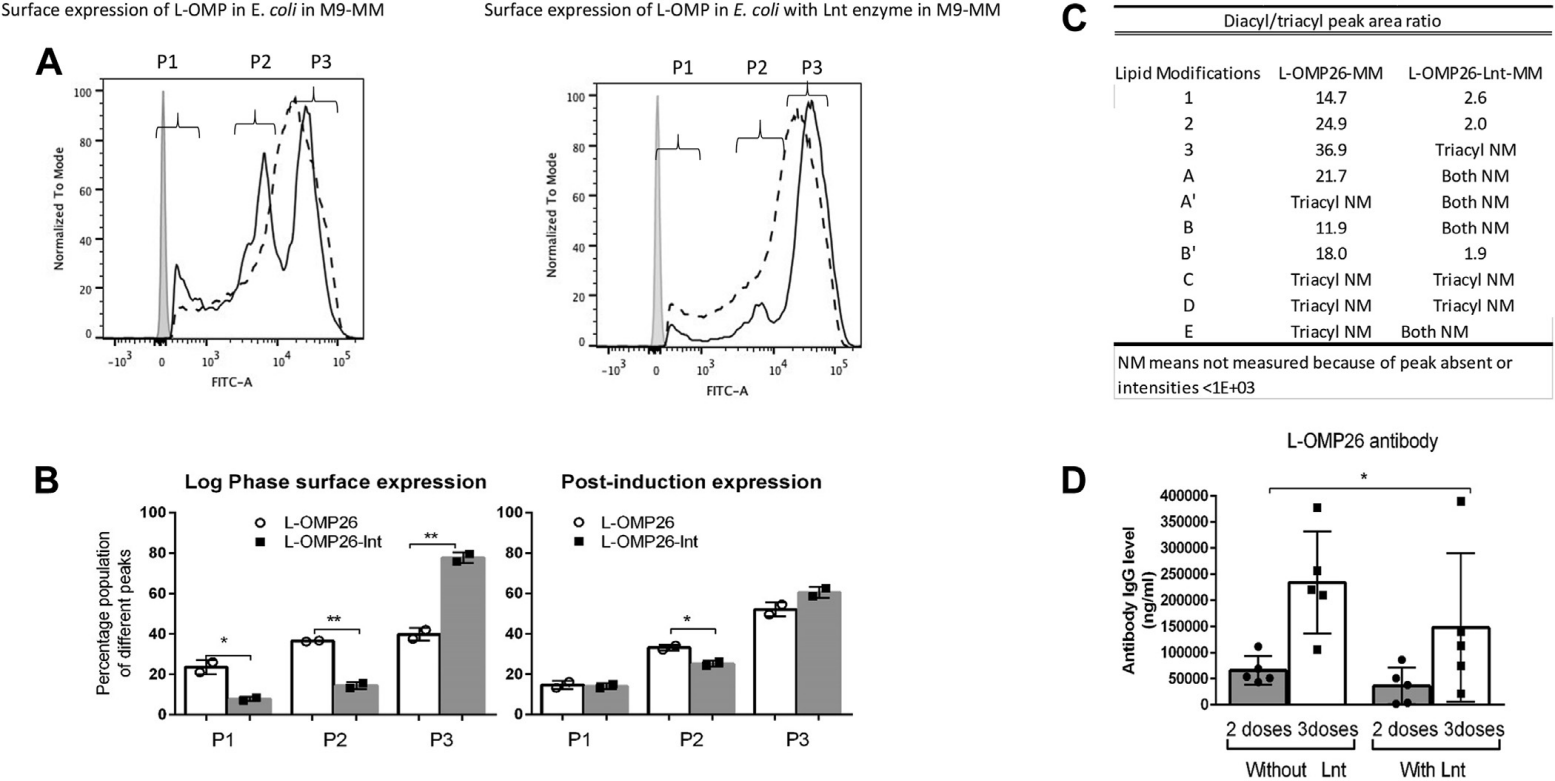
**Surface expression of L-OMP26 without and with Lnt coexpression.***A*, change in expression determined by flow cytometry of L-OMP26 on the surface of *Escherichia coli* grown without and with Lnt co-expression at log phase (*solid line*) and postinduction phase (*dotted line*). *Leftmost gray* peak is unstained control. Three peaks (P1, P2, and P3) were assigned at log phase. P1 corresponds to diacylated L-OMP26 with signal sequence still attached; P2 corresponds to diacylated L-OMP26 without signal sequence and P3 corresponds to triacylated L-OMP26 (this is same figure as shown in Figure 2*Aright panel*). *B*, population density area of P1, P2, and P3 peaks shown as proportion expression. *p*-values by *t* test. *C*, mass spectrometry analysis of different lipid modifications and plotted population density areas of peaks of diacyl *versus* triacyl L-OMP26 grown without and with Lnt co-expression. *D*, comparison of IgG levels after two doses and three doses of L-OMP26 vaccinations when *E. coli* were grown in different conditions. *p*-value by Mann-Whitney test after three doses. ∗*p* < 0.05; ∗∗*p* < 0.01; and ns = not significant.

As shown in Figure 1, Lnt enzyme promotes the transition from diacylated lipoprotein to triacylated lipoprotein. Figure 5*A* shows the surface expression of L-OMP26 in *E. coli* with and without Lnt enzyme co-expression. As described above, the P1 peak is presumed to correspond to L-OMP26 with the attached signal sequence, P2 to diacylated L-OMP26, and P3 to triacylated L-OMP26. The population density area for each of these three peaks was plotted in Figure 5*B*, indicating the percentage of the population corresponding to each OMP26 form. The two sets up bars (white and gray) correspond to L-OMP26 expressed with and without co-expression of Lnt in *E. coli* grown to log phase (before induction with IPTG) or post-IPTG induction. Cells grown to log phase show a decrease in the P1 (*p* < 0.05) and P2 (*p* < 0.01) populations with the addition of Lnt co-expression (Fig. 5*B*). In contrast, the P3 (triacylated) population increased (*p* < 0.01) with Lnt co-expression (Fig. 5*B*). After induction, we observed no significant difference in the P3 populations with or without Lnt co-expression, but we did measure a decrease in the P2 population (*p* < 0.05) with Lnt co-expression.

Similar to Table 1, MS/MS mass spectroscopy was employed to determine the ratio of diacylated/triacylated L-OMP26 from *E. coli* grown with and without Lnt co-expression. The MS data suggest that L-OMP26 was predominantly diacylated without Lnt co-expression but predominantly triacylated with Lnt co-expression (Fig. 5*C*). MS/MS spectra showing modification B′ of L-OMP26 from *E. coli* grown with and without Lnt is shown in Fig. S4. To test if the lipid modifications impacted immunogenicity, anti-OMP26 IgG antibody levels were measured at days 22 and 35 (Fig. 3*A*) after vaccination with two formulations of L-OMP26 (expressed in *E. coli* without and with Lnt co-expression) (Fig. 5*D*). L-OMP26 with enhanced triacylation induced significant lower (∼2 fold lower; *p* < 0.05) IgG antibody levels than predominantly diacylated L-OMP26 (Fig. 5*D*). Both L-OMP26 formulations, expressed with and without Lnt, induced higher (*p* < 0.001) antibody IgG levels than NL-OMP26 (data not shown). In addition, higher CCL5 levels occurred with predominant diacylated L-OMP26 (*E. coli* grown in M9-MM) compared to triacyl-enriched L-OMP26 (*E. coli* grown in Lnt-M9-MM) as shown in Fig. S5.

### Impact of acylation status on TLR2-TLR1 *versus* TLR2-TLR6 stimulation

**Figure 6:**
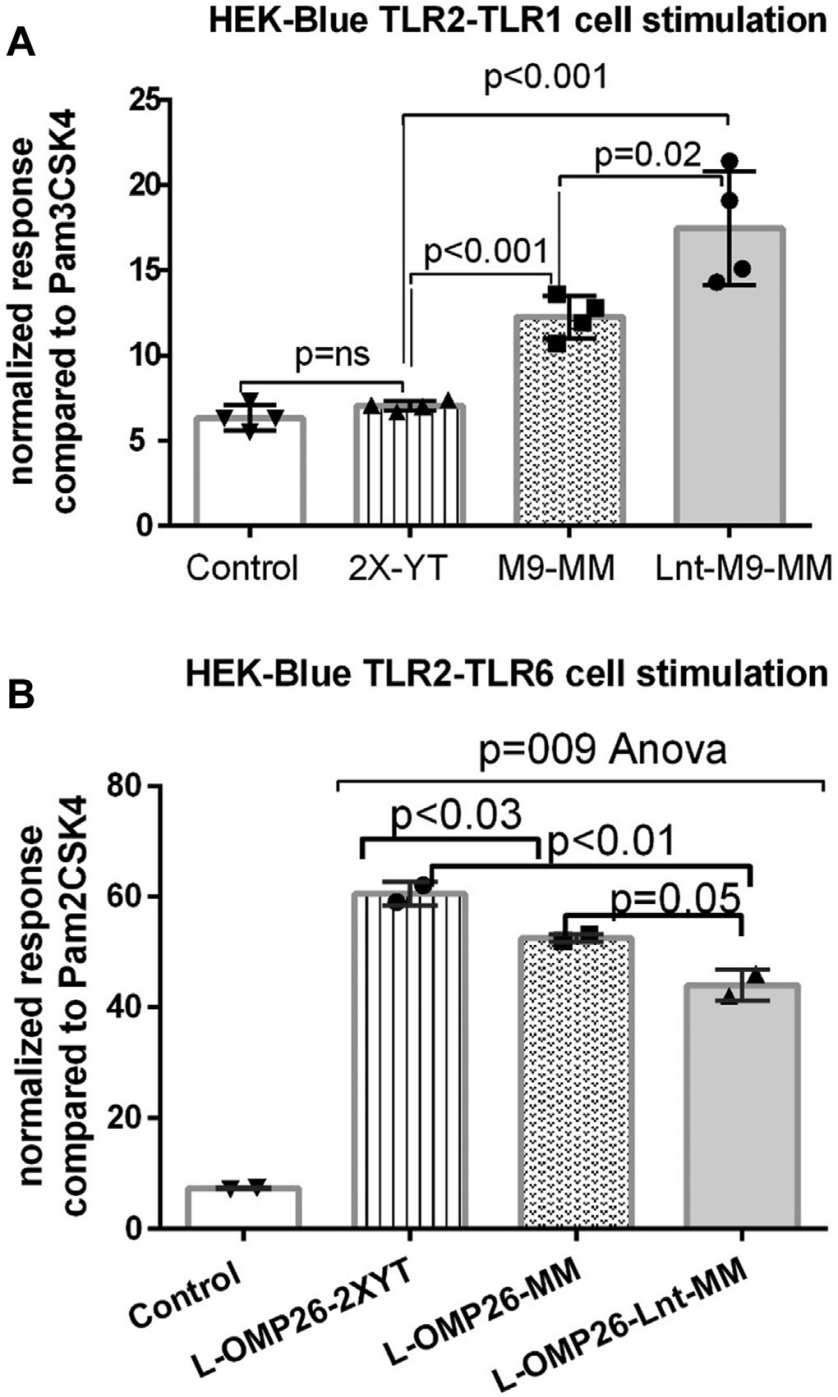
**HEK-Blue hTLR2-TLR1 and hTLR2-TLR6 cell line stimulation by L-OMP26.** After *Escherichia coli* were grown in 2XYT media, M9 minimal media (M9-MM), or M9-MM with Lnt co-expression. Y-axis displays normalized absorbance values of positive control Pam3CSK4 (*A*) and Pam2CSK4 (*B*). Mean with SD is shown along with individual points. *p*-value by ANOVA with multiple comparison correction of protein constructs with each other and control (No protein). *A*, stimulation of hTLR2-TLR1 cell line occurs when L-OMP26 was expressed as a triacyl construct. *B*, stimulation of hTLR2-TLR6 cell line occurs when L-OMP26 was expressed as a diacyl construct. Note: PAM3CSK4, the positive control for determining triacyl formulations, stimulated HEK-Blue hTLR2-TLR6 cells, responsive to diacyl lipids; therefore, the HEK-Blue hTLR2-TLR6 cell line cannot be used to distinguish diacyl and triacyl lipidation. *p*-values determined by ANOVA with multiple comparisons. TLR, toll-like receptor.

During stimulation with lipidated proteins, TLR2 forms a heterodimer with coreceptors TLR1 or TLR6, depending upon triacylation or diacylation of the lipid, respectively (31). Two cell lines (HEK-Blue hTLR2-TLR1 and HEK-Blue hTLR2-TLR6 cells) were stimulated with L-OMP26 purified from *E. coli* grown in 2X-YT media, M9-MM, and M9-MM with Lnt co-expression (Lnt-M9-MM) to determine the effect of lipidation status on cell stimulation. L-OMP26 purified from *E. coli* grown in 2X-YT media did not stimulate hTLR2-TLR1 cells (Fig. 6*A*), consistent with our original prediction of low levels of triacyl lipidation. L-OMP26 purified from *E. coli* grown in M9-MM stimulated hTLR2-TLR1 cells to 13% compared to positive control (Pam3CSK4), consistent with low levels of triacyl lipidation (Fig. 6*A*). L-OMP26 co-expressed with Lnt stimulated hTLR2-TLR1 cells to 17% of positive control higher than both previous L-OMP26 versions (*p* < 0.001 and *p* = 0.02, respectively). We hypothesize that triacyl lipidation of L-OMP26 was low in all of the three of these L-OMP26 preparations.

All L-OMP26 preparations (2X-YT media, M9-MM, and with Lnt co-expression) strongly stimulated hTLR2-TLR6 cells along with Pam2CSK4-positive control, consistent with diacyl lipidation (Fig. 6*B*). The triacyl positive control (Pam3CSK4) also stimulated the hTLR2-TLR6 cells similar to Pam2CSK4 control; therefore, responses could reflect either triacyl- or diacyl-lipidated forms of L-OMP26. Considering the higher stimulation with hTLR2-TLR6 cells *versus* hTLR2-TLR1 cells (Fig. 6*B*), we interpreted the result to reflect predominant diacyl lipidation of L-OMP26. Among the preparations, L-OMP26 from *E. coli* grown in 2X-YT media stimulated a higher (∼60%) response from hTLR2-TLR6 cells than L-OMP26 from *E. coli* grown in M9-MM (*p* < 0.03) or M9-MM with Lnt co-expression (*p* < 0.01). L-OMP26 with Lnt co-expression showed the lowest hTLR2-TLR6 stimulation (40%), consistent with less diacylation.

### Characterization of L-OMP26φNL-P6 fusion protein

**Figure 7:**
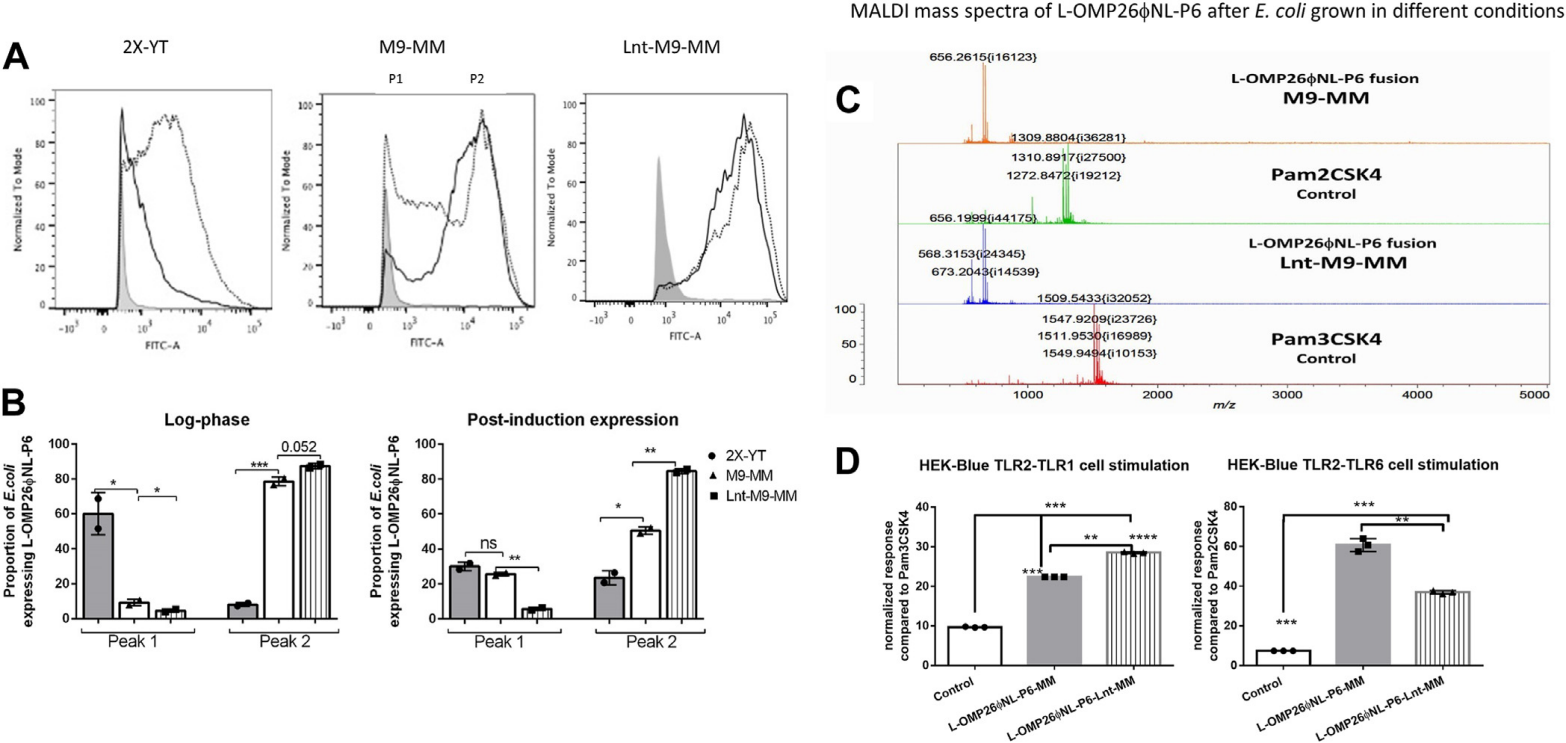
**Surface expression of L-OMP26φNL-P6 without and with Lnt coexpression.***A*, change in expression of L-OMP26φNL-P6 on the surface of *Escherichia coli* grown without and with Lnt co-expression at log phase (*solid line*) and postinduction phase (*dotted line*). *Leftmost gray* peak is unstained control. Two peaks (P1 and P2) were assigned at log phase. P1 corresponds to diacylated L-OMP26φNL-P6 with signal sequence still attached; P2 corresponds to diacylated L-OMP26φNL-P6 without signal sequence. P3 corresponding to triacylated L-OMP26φNL-P6 was not observed or hidden under P2 area. *B*, population density area of P1 and P2 peaks shown as proportion expression. *p*-values by *t* test. *C*, mass spectrometry analysis of different lipid modifications and plotted population density areas of peaks of diacyl *versus* triacyl L-OMP26φNL-P6 grown without and with Lnt co-expression. *D*, comparison of HEK-Blue hTLR2-TLR1 and hTLR2-TLR6 cell line stimulation by L-OMP26φNL-P6 after *E. coli* were grown in M9 minimal media (M9-MM) or M9-MM with Lnt co-expression. *p*-values determined by Anova with multiple comparisons. ∗*p* < 0.05; ∗∗*p* < 0.01; ∗∗∗*p* < 0.001 ∗∗∗∗*p* < 0.0001 and ns = not significant. NL, nonlipidated; TLR, toll-like receptor.

Figure 7*A* shows the surface expression of fusion protein L-OMP26φNL-P6, based on flow cytometry results, purified from *E. coli* cultures grown in 2X-YT media, M9-MM, and M9-MM media with Lnt co-expression. We propose that peak P1, with the lowest FITC labeling, corresponds to diacylated L-OMP26φNL-P6 with the attached signal sequence and the broad P2 peak corresponds to diacylated lipoprotein L-OMP26φNL-P6 without signal sequence. Peak P3, corresponding to triacylated product, is missing in the fusion protein construct or it is under and nondistinguishable from the broad P2 peak. The density areas of P1 and P2 were plotted (Fig. 7*B*). In log phase, a decrease of P1 occurred when *E. coli* were grown in M9-MM and M9-MM with Lnt co-expression (*p* < 0.05) compared to when *E. coli* were grown in 2X-YT media (Fig. 7*B*).

The P2 peak was small in log phase–harvested *E. coli* in 2X-YT media but increased when *E. coli* were grown in M9-MM or M9-MM with Lnt co-expression (*p* < 0.001). The result was consistent with the possibility that the increased size of the fusion product might hinder export to the surface of *E. coli*. After IPTG induction, the P1 peak (presumably L-OMP26φNL-P6) was lowest when *E. coli* were grown in M9-MM with Lnt co-expression and significantly lower compared to when *E. coli* were cultured without Lnt (*p* < 0.01), consistent with mature lipidated protein expression. The P2 peak was highest in Lnt-M9-MM than in M9-MM (*p* < 0.01) and lowest in 2X-YT media than in M9-MM (*p* < 0.05).

The MALDI-TOF mass spectra of L-OMP26φNL-P6 fusion grown with and without Lnt co-expression along with diacyl (Pam2CSK4) and triacyl (Pam3CSK4) controls is shown in Figure 7*C*. Comparison of MALDI spectra peak intensities for L-OMP26φNL-P6 grown with or without Lnt co-expression in M9-MM are shown in Table S2. Ratios of peak intensities are shown in Table S2, indicating Lnt enhances triacylation.

Figure 7*D* shows the comparison of stimulations of hTLR2-TLR1 and hTLR2-TLR6 cells using L-OMP26φNL-P6 expressed in *E. coli* (M9-MM) with and without Lnt co-expression. L-OMP26φNL-P6 grown in M9-MM stimulated hTLR2-TLR1 cells to 20% of positive control (Pam3CSK4), consistent with low levels of triacyl lipidation (Fig. 7*D*). L-OMP26φNL-P6 with Lnt co-expression stimulated hTLR2-TLR1 cells to 26% of positive control, an increase (*p* < 0.01) compared to M9-MM–grown *E. coli*, consistent with increased triacylation of L-OMP26φNL-P6 when co-expressed with Lnt. L-OMP26φNL-P6 stimulated hTLR2-TLR6 cells to 58% of positive control (Pam2CSK4), consistent with diacyl lipidation (Fig. 7*D*). L-OMP26φNL-P6 expressed in *E. coli* with Lnt co-expression stimulated hTLR2-TLR6 cells to 38% of positive control, reflecting a decrease in diacyl lipidation compared to L-OMP26φNL-P6 without Lnt co-expressed levels (*p* < 0.01).

We also determined the effect of different pH conditions on the accumulation of diacylated or triacylated L-OMP26φNL-P6 at pH values of 5, 6, 7, and 8 (Fig. S6). At pH 5 and 6, *E. coli* cells produced more diacylated L-OMP26, represented by peak P5 in the post–logarithmic-growth phase. The maximum peak intensity for P5 was observed at pH 5 (77% population). The percentage of diacylated L-OMP26φNL-P6 at pH 6.0 was higher than the percentage at pH 7 (61% at pH 6 *versus* 25% at pH 7; *p* < 0.01) and only slightly higher at pH 8 (30%). In contrast, M9 MM medium buffered to pH 7 yielded higher (*p* < 0.01) triacylated L-OMP26φNL-P6 (P6 peak 55% compared to 20% at pH 6) in the post–logarithmic-growth phase, with no further increase in P5 peak intensity at pH 8, consistent with the pH result patterns of L-OMP26.

L-OMP26φNL-P6 from *E. coli* grown in M9-MM media with and without co-expressed Lnt stimulated hTLR2-TLR1 cells (Fig. S7). L-OMP26φNL-P6 from M9-MM stimulated hTLR2-TLR1 cells to 21% of positive control (Pam3CSK4), consistent with lower levels of triacyl lipidation (Fig. S7). L-OMP26φNL-P6 co-expressed with Lnt showed enhanced hTLR2-TLR1 stimulation (32% of positive control, *p* < 0.001), consistent with enhanced triacylation with additional Lnt co-expression.

## Discussion

The goal of this study was to characterize the conversion of a naturally NL membrane protein of *H. influenzae*, OMP26, into a lipidated protein, L-OMP26, to enhance its immunogenicity as a vaccine candidate. We also characterized an L-OMP26φNL-P6 fusion protein to determine whether or not a larger fusion protein would impact the lipid modification. We found that recombinantly expressed L-OMP26 and L-OMP26φNL-P6 fusion were predominantly produced in diacylated form, unlike most naturally lipidated proteins from Gram-negative bacteria, which are typically triacylated (1, 29). We conducted experiments to optimize mature lipoprotein expression with the hypothesis that diacylated lipoprotein vaccines would cause less reactogenicity and better immunogenicity compared to triacylated lipoproteins; our experimental results corroborated this hypothesis. To our knowledge, this is the first study of its kind for *E. coli* of *H. influenzae proteins*, and there has been only one prior study of a lipoprotein vaccine construct design that explored concepts of preferential diacyl *versus* triacyl lipidation in *S. aureus* (8).

In *E. coli*, the biosynthetic pathway of making a triacylated outer membrane lipoprotein starts with the diacylated version of the lipoprotein in the inner membrane. A third lipid is added to produce the triacylated lipoprotein before translocation to the outer membrane. We explored environmental methods to alter the diacyl/triacyl ratios of OMP26 using nutrient-rich and nutrient-poor media to culture *E. coli*, which recombinantly expressed our vaccine candidates. We employed flow cytometry and mass spectrometry to identify and quantify lipoprotein expression and determined that L-OMP26 and L-OMP26φNL-P6 fusion expression and lipidation status changed based on the type of media used to culture *E. coli* during recombinant expression of the vaccine candidates. Nutrient poor growth media proved superior for the expression of mature lipoproteins compared to nutrient-rich media. Tseng *et al* (31) reported a similar observation, consistent with the notion that nutrient-deficient growth media reduces the level of protein synthesis, providing the benefit of not overloading the lipidation pathway and translocation machinery for recombinantly expressed lipoproteins (32, 33).

Growth phase also impacted the maturation of lipoproteins during recombinant production in *E. coli.* In log phase, much of the protein product was recovered with the signal sequence still attached, consistent with an immature lipoprotein. After IPTG induction, in the preferred nutrient poor growth media, we observed no difference in diacyl lipoprotein product, but an increase in triacyl product was observed. We also determined that diacyl lipoprotein products were optimally produced by *E. coli* when grown in nutrient poor media at pH 6 and triacyl lipoprotein products at pH 7. Kurokowa *et al* (8) had previously described and demonstrated environment-mediated accumulation of diacyl lipoproteins in *S. aureus*.

We found that higher lipidation status resulted in higher levels of cytokine IL8 and chemokine CCL5 (RANTES), which may be associated with higher reactogenicity. Higher antibody levels to L-OMP26 were induced when there were higher levels of lipidation, which is consistent with other reports that lipidation increases immunogenicity of proteins (19, 34). Higher CCL5 stimulation occurred with predominant diacylated L-OMP26 vaccinations compared to triacyl-enriched L-OMP26. CCL5 has been shown to enhance/recruit human immature dendritic cells (35) and can act as an anti-inflammatory chemokine in some settings. CCL5 also is closely linked to its actions in regulating T-cell performance (36).

Using these lipoproteins and LC/MS mass spectra analysis, we successfully identified two groups of N-terminally lipidated (diacyl or triacyl) molecules that differ by one fatty acid unit, as described previously (37). All L-OMP26 preparations strongly stimulated hTLR2-TLR6 cells, consistent with predominant diacyl lipidation. To study the effects of increased triacylation of L-OMP26, we co-expressed the *lnt* gene on a separate plasmid in *E. coli* along with a plasmid containing the *omp26* gene. Co-expression of Lnt resulted in increased expression of mature diacylated L-OMP26 protein on the *E. coli* cell surface and an increased proportion of triacyl L-OMP26 lipoprotein. L-OMP26 with enhanced triacylation induced lower IgG antibody levels than predominantly diacylated L-OMP26 but higher antibody levels than NL-OMP26. These results suggest that predominantly diacylated L-OMP26 would be a preferred vaccine candidate.

Stimulation of TLR2-TLR6, which occurred using all of the diacylated L-OMP26 formulations, may be preferred over TLR2-TLR1 stimulation. Nguyen *et al* (19) showed that the long-chain N-acylated Lpp protein of *Staphylococcal* spp. that was recognized by TLR2–TLR1 receptors silenced innate and adaptive immune responses, while the short-chain N-acetylated Lpp, that was recognized by TLR2–TLR6 receptors, boosted it. Their observation was based on commensal and virulent species, but here we show for the first time that the same protein with different levels of acylation moieties can impact immune response after vaccination of mice.

We also investigated whether adding an NL second protein by fusion to L-OMP26 would affect its lipidation properties. In prior work, we reported that L-OMP26φNLP6 fusion enhanced the immune response to both the L-OMP26 and fused NL-P6 proteins after vaccination of mice (26). In those experiments, L-OMP26φNL-P6 fusion had higher triacylation levels (∼25%) than L-OMP26 alone (15%). In this current study, L-OMP26φNL-P6 fusion, when produced in *E. coli* grown in specific media and pH conditions and with Lnt co-expression, yielded a similar lipidation profile to nonfusion L-Omp26 protein under those same conditions. Vaccination with L-OMP26φNL-P6 fusion and nonfusion L-OMP26 protein induced similar antibody responses to L-OMP26.

Our study has some limitations. In the flow cytometry surface expression experiments, assumptions were based on the known biology of lipoprotein expression in *E. coli* cell membranes to assign the peaks as representing variations of the lipoprotein constructs. However, we did not confirm that the peaks correspond to the lipoprotein populations described. It is possible that some of the differences in low intensity of fluorescent peaks could be due to misfolded proteins as shown by Nicchi *et al* (38), rather than different acylated peaks as we described. LC/MS analysis of L-OMP26 with Lnt co-expression was consistent with identifying higher triacylation of the construct, as expected, compared to the extent of triacylation that occurred when L-OMP26 expression in *E. coli* grown in M9-MM media. Results from our HEK2 cell analysis led us to similar conclusions. Specifically, HEK2-hTLR2-TLR1 cells that are stimulated by triacyl lipoproteins responded more effectively to L-OMP26 produced with Lnt co-expression than to L-OMP26 expressed in *E. coli* grown in M9-MM media, although fold-differences between the two testing methods were not the same. Experiments to compare reactogenicity and immunogenicity after vaccination with L-OMP26 grown in two different media conditions were limited by the detection of only IL-8 and CCL5 among the 10 cytokines/chemokines tested. In assessing the impact of Lnt enzyme on the surface expression of L-OMP26 in *E. coli* and on acylation modifications and immunogenicity of L-OMP26, the enhancement in triacylation was relatively small, although statistically significant. With naturally lipidated P6 protein of *H. influenzae*, we previously found that hTLR2-TLR1 cells were stimulated to ∼60 to 80% of positive control (26). In assessing the impact of acylation status on TLR2-TLR1 *versus* TLR2-TLR6 stimulation, the triacyl positive control also stimulated the TLR2-TLR6 cells; therefore, we were unable to indisputably define the impact of triacyl *versus* diacyl lipid constructs. The experiments to characterize surface expression on *E. coli* and on acylation modifications of L-OMP26 fused with NL P6 *H. influenzae* protein L-OMP26φNL-P6 fusion had the same limitations as those referenced above for L-OMP26.

In conclusion, in this study, we successfully added a lipid moiety to OMP26, a native NL protein of *H. influenzae*. We characterized recombinantly expressed L-OMP26 from *E. coli* and fusion protein L-OMP26φNL-P6 and described several strategies to alter the diacylated/triacylated lipoprotein ratios, including changing pH, culture media, and co-expression with Lnt enzyme. Finally, we showed that the diacyl and triacyl L-OMP26 protein formulations differ markedly in their immune-stimulatory activity, with diacylated L-OMP26 stimulating higher adaptive immune responses than triacylated L-OMP26. Our findings have general applicability to the field of biological chemistry in vaccine development. Lipidation of other candidate proteins that are not naturally lipidated of *H. influenzae* or other bacteria could follow our experimental approaches. Preferential preparation of predominantly diacylated lipoproteins may be pursued to optimize immunogenicity and potentially reduce reactogenicity.

## Experimental procedures

### Lnt cloning

Plasmid, pACYC184, was isolated from *E. coli*/pACYC184 (ATCC #37033) using the Monarch plasmid miniprep kit (catalog # T1010S, New England Biological (NEB)) and digested with EcoR1 and Sca1 (NEB). The *lnt* gene was isolated from *E. coli* DH5 genomic DNA *via* standard PCR using a PCR kit (PR1001-1000, ACCURIS) and Lnt-EcoR1 primer 5′GATATAGAATTCTAATAAAACCGAAACTGGATAGATAACTA & Lnt-Sca1 primer 5′ GATATAAGTACTTTATTTACGTCGCTGACGCAGAC, synthesized by Integrated DNA Technologies. PCR reaction components were removed using the Monarch PCR & DNA cleanup kit (catalog # T1030S, NEB), and the DNA was digested with EcoR1 and Sca1 (NEB). Both the digested *lnt* gene and pACYC184 DNA were electrophoresed on a 1.25% agarose gel, and the respective DNA fragment bands were isolated from the gel using a Monarch DNA gel extraction kit (catalog # T1020S NEB) and ligated using T4 DNA ligase (catalog # M0202S, NEB). The ligated DNA was transformed into high efficiency 5-alpha–competent *E. coli* cells (catalog # C2987H, NEB) as recommended by the manufacturer. The transformed cells were spread onto 2X-YT plates containing 10 μg/ml tetracycline. Tetracycline-resistant colonies were patched onto 2X-YT plates containing 25 μg/ml chloramphenicol. Plasmid DNA was isolated using the Monarch plasmid miniprep kit from colonies that were confirmed to be tetracycline-resistant and chloramphenicol-sensitive. Restriction enzyme digestion using EcoR1 and Sca1 was done to verify the *lnt* insert. DNA sequence analysis (GeneWiz) confirmed the insert of the *lnt* gene and its predicted amino acid sequence.

### Cotransformation of L-OMP26 and PAC-Lnt plasmids into competent cells

The pet21a plasmid DNA containing *L-omp26* gene and pACYC184 containing *lnt* gene were cotransformed into *E. coli* C43 (DE3) chemically competent cells (catalog # 60446-1, Lucigen), following the manufacturer’s protocol using 122 ng of *L*-*omp26* plasmid DNA and 350 ng of Lnt plasmid DNA. Transformed colonies were selected with LB media plates containing both 100 μg/ml ampicillin and 10 μg/ml tetracycline. Colonies were selected and tested by PCR for confirmation of transformation.

### *E. coli* growth and protein expression containing L-OMP26

Chemically competent *E. coli* strains C43 (DE3) (Lucigen) containing transformed plasmids 1) *L-omp26*, 2) *L-omp26* + *lnt*, 3) *L-omp26φNL-p6*, and 4) *L-omp26φNL-p6*+ *lnt* were used for expressing the L-OMP26 proteins and fusions. Precultures were all grown overnight in 2X-YT media with 100 μg/ml ampicillin and addition of 10 μg/ml tetracycline to the Lnt co-expression containing batch. The precultures were then used to inoculate larger volumes (1–3 L) of 2X-YT media or M9 minimal media (M9-MM) (9 mM NaCl, 22 mM KH_2_PO_4_, 47.8 mM Na_2_HPO_4_∙7H_2_O, 19 mM NH_4_Cl, 2 mM MgSO_4_, 0.1 mM CaCl_2_, 0.4% glucose) with the same antibiotics used for precultures (2X-YT media was washed and replaced with M9-MM prior to M9-MM inoculations). The cells were either grown in 2X-YT media or M9-MM (L-OMP26 and L-OMP26-Lnt co-expression batch). Cells were grown at 37 °C until reaching an A_600_ ∼0.6 prior to the induction of protein expression with 0.4 mM IPTG, overnight at 30 °C in M9-MM. In 2X-YT media, cells were grown for 3 to 4 h after IPTG induction. Cells were then harvested *via* centrifugation at 10,000 rpm for 10 min and pellets were frozen at −20 °C for further purification.

### Flow cytometry analysis for surface expression

To observe the expression of L-OMP26 and L-OMP26φNL-P6 fusion on the surface of *E. coli* containing different plasmids, *E. coli* were grown in smaller volume (10–20 ml) under different conditions of pH in M9-MM. *E. coli* were sedimented and resuspended in PBS after growth. Bacterial cells (2 × 10^8^) were incubated with sera containing anti-NL-OMP26–specific antibody for 1 h at room temperature. After washing, bacteria were incubated for 30 min at room temperature in the dark with FITC-coupled goat anti-mouse IgG. After washing, bacterial cells were run on a BD-LSR II flow cytometry system (BD Biosciences), and 100,000 events were acquired for each sample. The data were analyzed with FlowJo Software V10 (https://www.flowjo.com).

### Recombinant lipidated protein purification

After protein expression, the frozen *E. coli* cells were thawed and suspended in lysis buffer (50 mM Tris/300 mM NaCl/100 μg/ml lysozyme/1 mM PMSF) and lysed by three cycles of sonication followed by incubation in a water bath at 37 °C for 20 to 30 min. The lysates were spun using ultracentrifugation at 41,100 g for 30 min at 4 °C. The supernatant containing cellular contents was removed and extraction buffer solution (1% triton-100/2% zwittergent 3-14/50 mM Tris/300 mM NaCl) was added to the pellet to extract the L-OMP26 and L-OMP26φNLP6 proteins. Lipidated OMP26 proteins were not extracting with triton-100 only, so zwittergent 3-14 detergent was used along with triton-100. DNase 1 at 5 μg/ml was added in solution and incubated for 10 min at room temperature, followed by sonication and water bath incubation at 37 °C for 30 min. Ultracentrifugation at 154,049*g* for 30 min at 4 °C was performed, and the supernatant containing extracted protein was saved and the extraction process repeated. The two extractions were combined for purification. Bio-Rad Profinity IMAC Ni-charged resin was used to purify extracted His-tagged lipidated OMP26 protein as described previously (26) using imidazole. Final purified lipidated proteins were exchanged in PBS buffer along with 0.05% zwittergent 3-14. Proteins were passed through a column for endotoxin removal (high capacity endotoxin removal spin column by Pierce, catalog # 88275) and stored at −80 °C for further experiments. Endotoxin levels were tested for each protein lot with Pierce chromogenic endotoxin quantitation kit (catalog# A39552S) before use in all experiments.

### *In vitro* HEK-Blue-hTLR2-TLR1, HEK-Blue-hTLR2-TLR6 cell stimulations

Stimulation of HEK-Blue-hTLR2-TLR1 (Invivogen, catalog # hkb-htlr21) and HEK-Blue-hTLR2-TLR6 (Invivogen, catalog # hkb-htlr26) with lipopeptides/lipoproteins results in activation of the TLR2/TLR1 or TLR2/TLR6 signaling pathway, respectively. Activation of TLR2/TLR1 or TLR2/TLR6 in turn activates the intracellular NF-kB and AP-1 (MyD88 pathway–dependent) signaling pathways, leading to an innate immune response. The HEK-Blue hTLR2-TLR1 cells were used as a reporter cell line to detect and quantitate triacylated lipoprotein/lipopeptide constructs that signal through TLR2/TLR1. The HEK-Blue-hTLR2-TLR6 was used as reporter cell line to detect and quantitate diacylated lipoprotein/lipopeptide constructs, which signals through the TLR2/TLR6 pathway. The phosphatase activity of secreted embryonic alkaline phosphatase, which is proportional to the level of stimulation, was quantitated using colorimetric assays according to a standard protocol. HEK-Blue-hTLR2-TLR1 and HEK-Blue-hTLR2-TLR6 cells (2.8 × 10^5^ cells/ml in 96-wells plate) were incubated individually with L-OMP26 (grown under different expression conditions) and Pam3CSK4 and Pam2CSK4 (a commercial TLR2/TLR1 and TLR2/TLR6 agonist standard, respectively) along with other controls (PBS and NL-OMP26) for 20 to 22 h at 37 °C. Each sample was tested in duplicate with different amounts of proteins (100 ng, 1 μg, and 10 μg/ml). The cell supernatant was diluted into QUANTI-Blue detection medium (InvivoGen #rep-qb1) and incubated at 37 °C for 1 to 3 h prior to measuring the absorbance at 620 to 655 nm for secreted embryonic alkaline phosphatase activity. In prior studies (26), we found that the triacyl positive control (Pam3CSK4) stimulated both the HEK-Blue-hTLR2-TLR1 and HEK-Blue-hTLR2-TLR6 reporter cell line; therefore, responses induced in HEK-Blue-hTLR2-TLR6 reporter cell line by our constructs may reflect either triacyl or diacyl lipids. We interpreted the results of the experiments, by comparing the quantitative read out from both cell lines to determine predominance of diacyl *versus* triacyl lipidation of L-OMP26 as previously reported (26).

### Mass spectrometry analysis for lipidation status of L-OMP26 and fusion L-OMP26φNL-P6

For mass spectrometry analysis, ∼10 μg of each protein was denatured using 1% SDS by heating at 98 °C for 10 min. Protein was precipitated using acetone and incubated at −20 °C for 3 h. After centrifugation (20,000*g*, 4 °C, 30 min), pelleted protein was washed gently with 500 μl methanol and air-dried briefly. Proteomics-grade trypsin (Cat#T6567, MilliporeSigma) was activated by dissolving in 50 mM Tris, pH 8.5 (0.25 mg/ml final concentration), and activated trypsin was added to each sample to reach a final enzyme:substrate ratio of 1:20. The samples were incubated at 37 °C for 6 h with constant shaking for proteolytic digestion followed by acidification with formic acid (1% final concentration) to inactivate trypsin and centrifuged at 20,000*g*, 4 °C, 30 min. The supernatant was carefully transferred to LC vials for LC-MS analysis.

LC-MS experiment: 100 ng of digested peptides were loaded into the LC-MS system. The system consisted of a Dionex Ultimate 3000 nano LC system, a Dionex Ultimate 3000 gradient micro LC system with a WPS-3000 autosampler, and an Orbitrap Fusion Lumos mass spectrometer (Thermo Fisher Scientific). Mobile phase A and B for nano LC were 0.1% formic acid (FA) in 2% acetonitrile (ACN) and 0.1% FA in 88% ACN. The nano LC gradient was 5 to 15% B for 10 min, 15%-75% B for 32 min, 75 to 97% for 20 min, and isocratic at 97% B for 60 min before equilibration to 5% B. The MS data was collected using 3-s maximal duty cycle time. The MS1 spectra were acquired in *m/z* range of 400 to 1500 at a resolution of 120,000 with automated gain control target of 500,000 and maximum injection time of 50 ms. The precursor ions were filtered using quadrupole (isolation window- 1.2 Th) and fragmented using high-energy collision dissociation at a normalized collision energy of 30%, and the dynamic exclusion was set to 45s ± 10 ppm. The MS2 spectra were acquired using IonTrap, with automated gain control target set to ‘standard’ and a maximum injection time of 50 ms.

### LC-MS spectra data processing

The raw data files were processed using Proteome Discoverer 2.2 (Thermo Fisher Scientific, https://www.thermofisher.com/us/en/home/industrial/mass-spectrometry/liquid-chromatography-mass-spectrometry-lc-ms/lc-ms-software/multi-omics-data-analysis/proteome-discoverer-software.html). Database searching was performed by matching the acquired MS/MS spectra against the theoretical spectra generated *in silico* from L-OMP26 protein sequence using SEQUEST HT engine. Peptides were identified using a false discovery rate of <1%. The ion current peaks for peptides of interest were manually extracted using theoretical m/z for each peptide modification and a mass tolerance of 10 ppm. The proportion of diacylated and triacylated peptides was determined using the extracted peak areas.

### MALDI-TOF experiment

MALDI-TOF MS was conducted using a Shimadzu Axima mass spectrometer in positive reflectron mode. The saturated α-cyano-4-hydroxycinnamic acid solution in a 30:70% H_2_O/ACN with 0.1% TFA solvent was used as a matrix. A thin layer of α-cyano-4-hydroxycinnamic acid matrix was prepared, and the trypsin-digested L-OMP26 protein samples were deposited on the matrix and MALDI MS/MS spectra were acquired. We used both diacyl and triacyl synthetic peptides [Pam2 (dipalmitoyl)-CSK4 and Pam3 (tripalmitoyl) CSK4] spectra to confirm samples could be analyzed with respect to organic solvent extraction and ionization. For MALDI experiment, we did not compare the efficiencies of ionization of the synthetic diacyl and triacyl synthetic peptides. Generally, major compounds are more effectively ionized and detected than minor compounds in the analysis. Therefore, the ratio of the diacyl form to the total amount was not calculated from the MALDI analysis here; only the difference in spectra were roughly indicated.

### Vaccination with proteins in mice for reactogenicity and immunogenicity

Inbred C57BL/6J mice were used with the approval and guidance of the Rochester Regional Animal Care and Use Committee. Six- to eight-week-old mice were equally distributed between males and females. Mice were vaccinated by intraperitoneal injection at week 0, 1, and 3 with 200 μl volume/animal (5 μg dose of each protein). Fifty to hundred microliters of blood was drawn 24 h after each injection from each mouse to determine cytokine profiles associated with reactogenicity. Blood was also drawn at week 5. At week 5, mice were challenged by *H. influenzae* strains as previously described (26, 39, 40). No additional adjuvant was used in immunization as we recently showed that the studied lipidated proteins were self-adjuvanting (26).

### Cytokine measurement for reactogenicity

For reactogenicity, cytokines in mouse sera were quantified using R&D Systems kit LXSAMSM-10 for CCL2/MCP-1, CXCL1/GRO alpha/KC/CINC-1 (IL-8 equivalent in mice), IFN-γ, IL-6, IL-17/IL-17A, CCL5/RANTES, CXCL2GRO beta/MIP-2/CINC-3, IL-1β, IL-10, and TNF-α by following instruction protocol and reagents provided in the kit. Briefly, mouse sera from 24 h postvaccination draws were diluted 1:1 with assay diluent and mixed with magnetic beads coated for the specific cytokines. Biotin-labeled antibody was used for secondary labeling followed by detection with streptavidin. After washing, beads were read on a Luminex Bio-Rad Bio-Plex200 instrument.

### Mouse anti-OMP26 serum IgG ELISA

Microplates (Microlon medium binding; Greiner Bio-One) were coated overnight at 4 °C with NL OMP26 protein at 1 μg/ml in carbonate buffer. For mouse IgG standard, one column was coated with goat anti-mouse IgG-Fc (Bethyl, cat#A90-131A) at 1 μg/ml. The coated plates were washed 5× with 1xPBS/0.1% Tween and blocked with 4% skim milk in 1xPBS/0.1% Tween. Mouse sera were diluted to 1:50,000 followed by 8, 2-fold serial dilutions and added to the washed plates. For the standard column, mouse reference serum (Bethyl, cat#RS10-101) was tested at 10 ng/ml followed by 8, 2-fold dilutions to be used as a relative quantitative measurement. Primary incubation was for 1 h at room temperature, followed by 5× washes and addition of horseradish peroxidase-secondary antibody at 1:5000 (Bethyl cat#A90-131P) and incubated for an additional hour. Plates were washed again before adding TMP substrate (KPL) and allowed to develop for 20 min at room temperature and stopped with the addition of 1M H_3_PO_4_. Plates were read at 450 nm. OMP26 IgG titers were approximated relative to the mouse standard column concentration.

### Statistical analysis

All statistical analyses were performed using GraphPad Prism version 6.0 (GraphPad Software, http://www.graphpad.com). A two-tailed *t* test on log-transformed data or a nonparametric Mann-Whitney test was used to compare two groups, as appropriate. One-way ANOVA with Bonferroni’s post-test was used to compare multiple groups in antibody measurement and cytokine values. Differences with a *p*-value < 0.05 were considered statistically significant.

## Data availability

All of the data is contained in the manuscript and supporting materials. Mass spectrometry data that support the conclusions in this study are available upon reasonable request.

## Supporting information

This article contains supporting information.

## Conflicts of interest

The authors declare that they have no conflicts of interest with the contents of this article.
