# Supplementary material for: Expression conditions and characterization of a novelly constructed lipoprotein intended as a vaccine to prevent human Haemophilus influenzae infections
Source: J Biol Chem. 2023 Jul 16;299(8):105031. doi: 10.1016/j.jbc.2023.105031 (PMC10407732; doi:10.1016/j.jbc.2023.105031)
Supplement: Supporting information [file mmc1.docx]

**Supporting Information:**


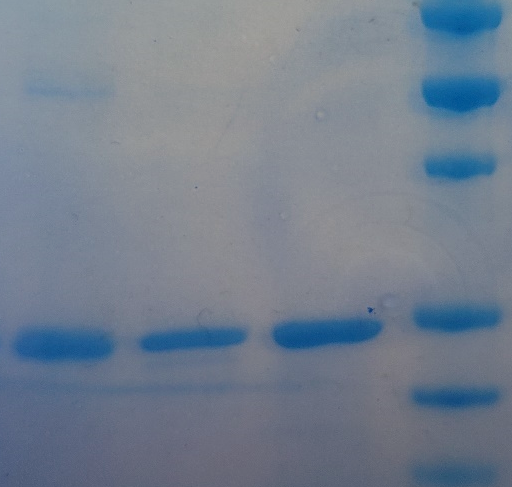

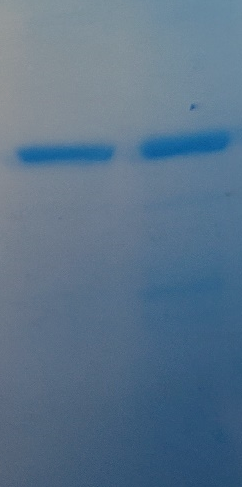


25kDa

37kDa

50kDa

20kDa

15kDa

2X-YT

M9 (Lnt)

M9

L-OMP26

L-OMP26φNL-P6

M9

M9 (Lnt)

Figure S1: SDS-PAGE characterization of purified Lipidated (L) OMP26 and L-OMP26ϕNL-P6 fusion along with molecular markers. Molecular weight bands are shown. The gel was run at the same time for L- OMP26 and L-OMP26ϕNL-P6 fusion, then spliced to put the molecular weight ladder lane in the middle.

Table S1: Endotoxin levels detected in the lipoprotein stocks and dose of endotoxin given to mice.

Modification 1 Charge +4

**Lipid modification #1 (Charge +4)**

**Triacyl L-OMP2 Diacyl L-OMP26**

**C16:0, C16:0, C16:1 C16:0, C16:1**

Triacyl

Diacyl

**M9 minimal media**

**2X-YT media**

Figure S2: Mass spectrometry analysis of L-OMP26 grown in M9-MM (top) and 2X-YT media (bottom) showing peak intensities of lipid modifications 1. (A) The peaks represent triacylated (left) and diacylated (right) L-OMP26 peptides. The peak area is highlighted in blue.


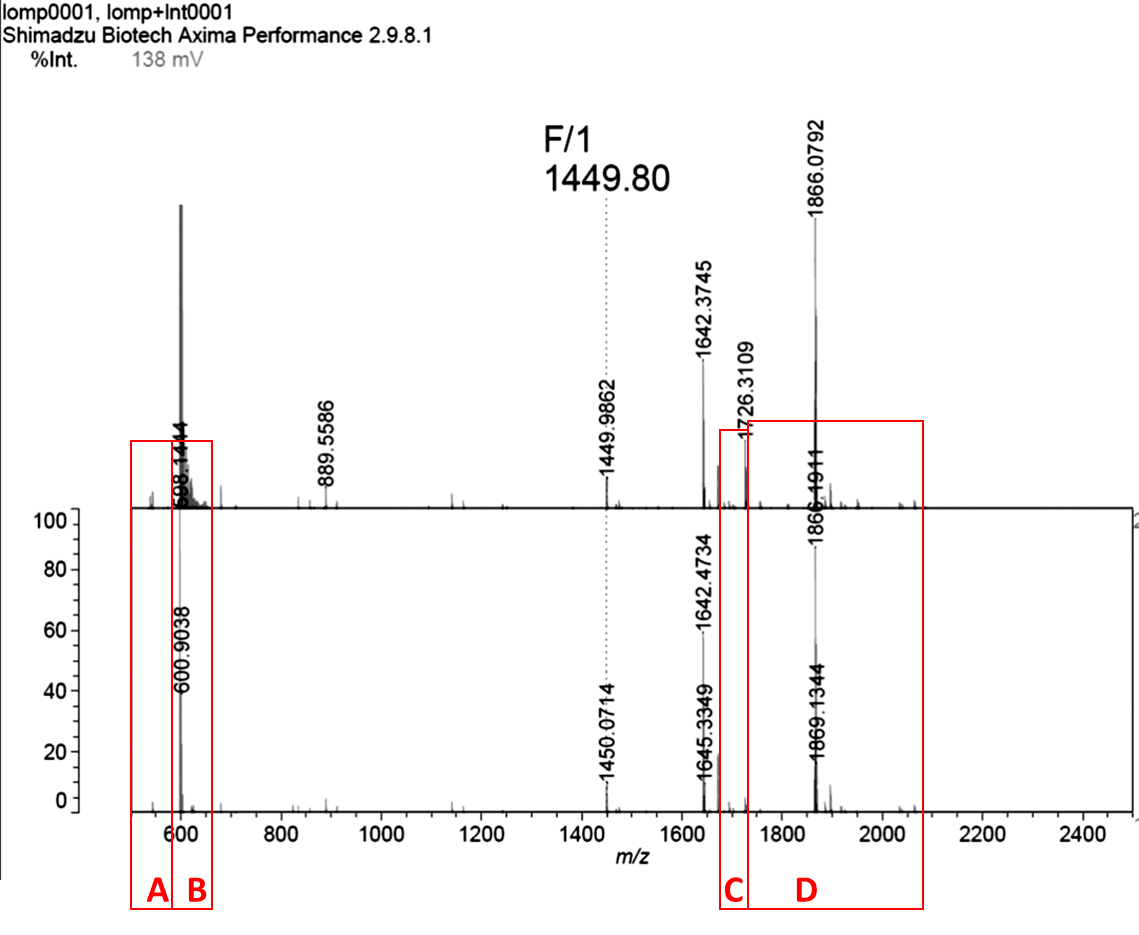


Inset A


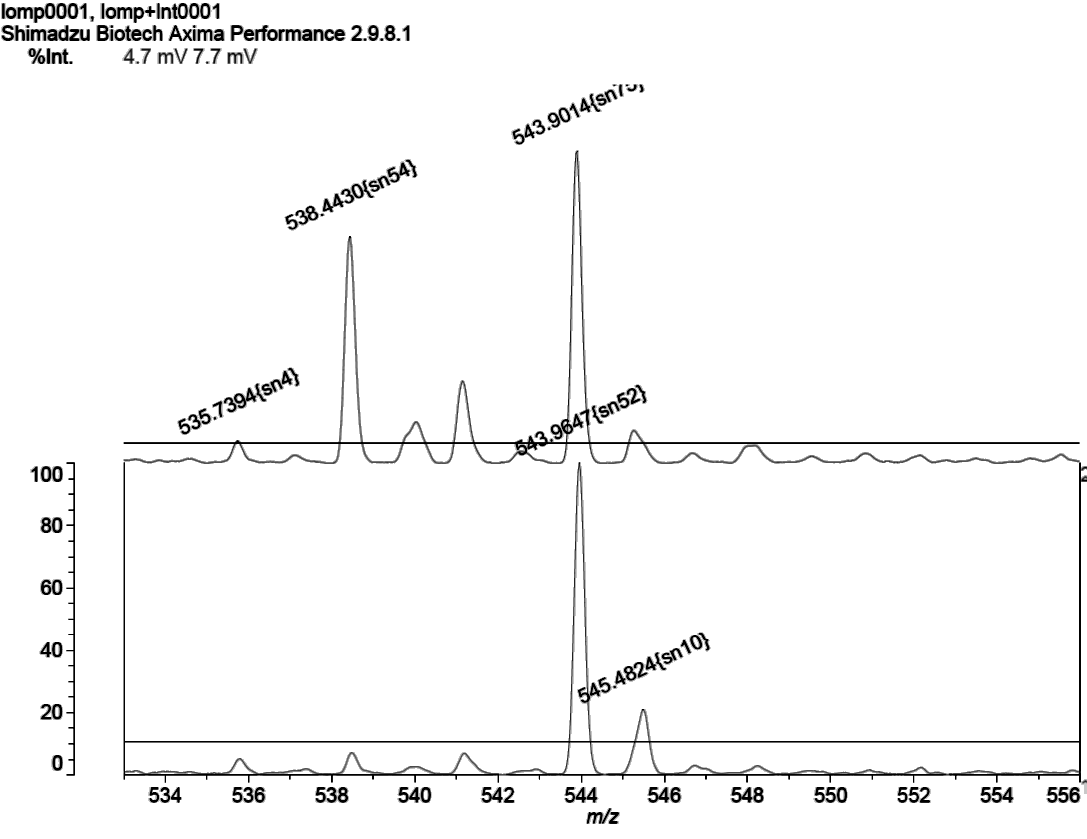


Inset B


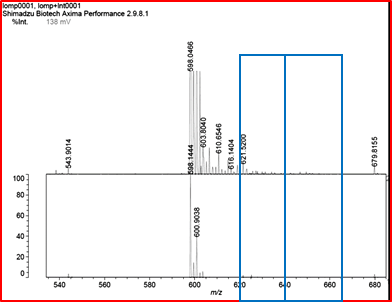


B1 B2

B1 B2


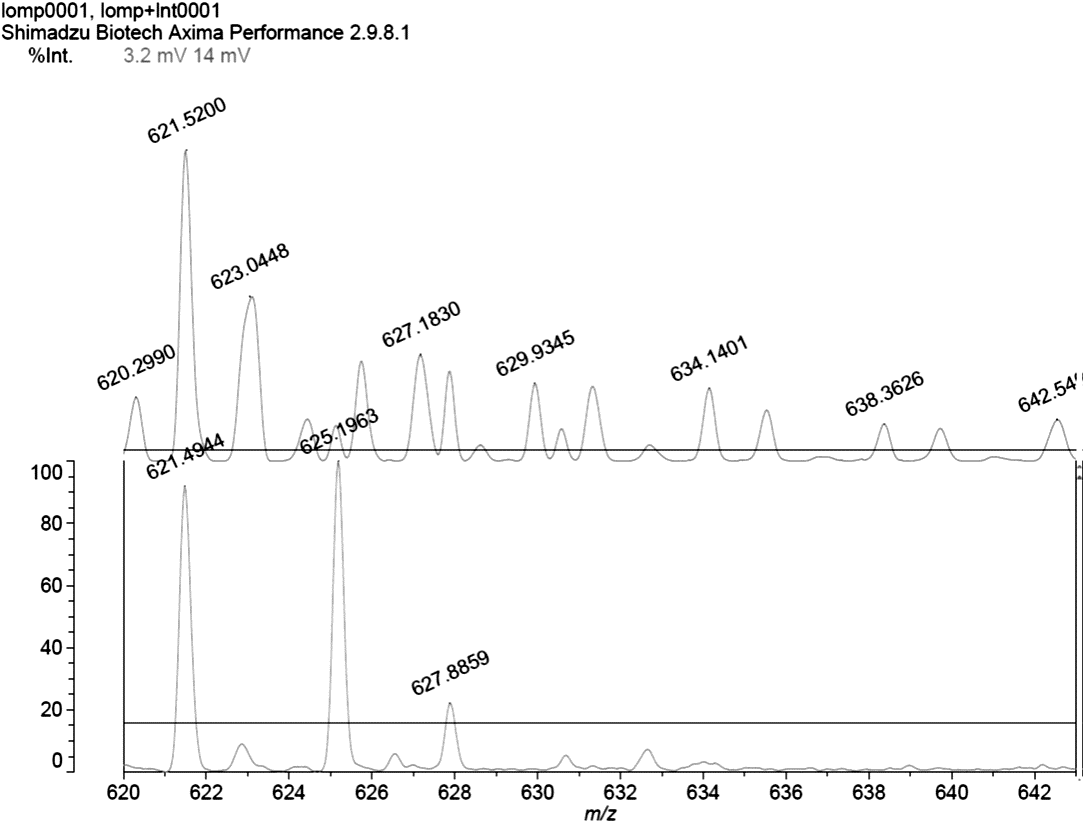

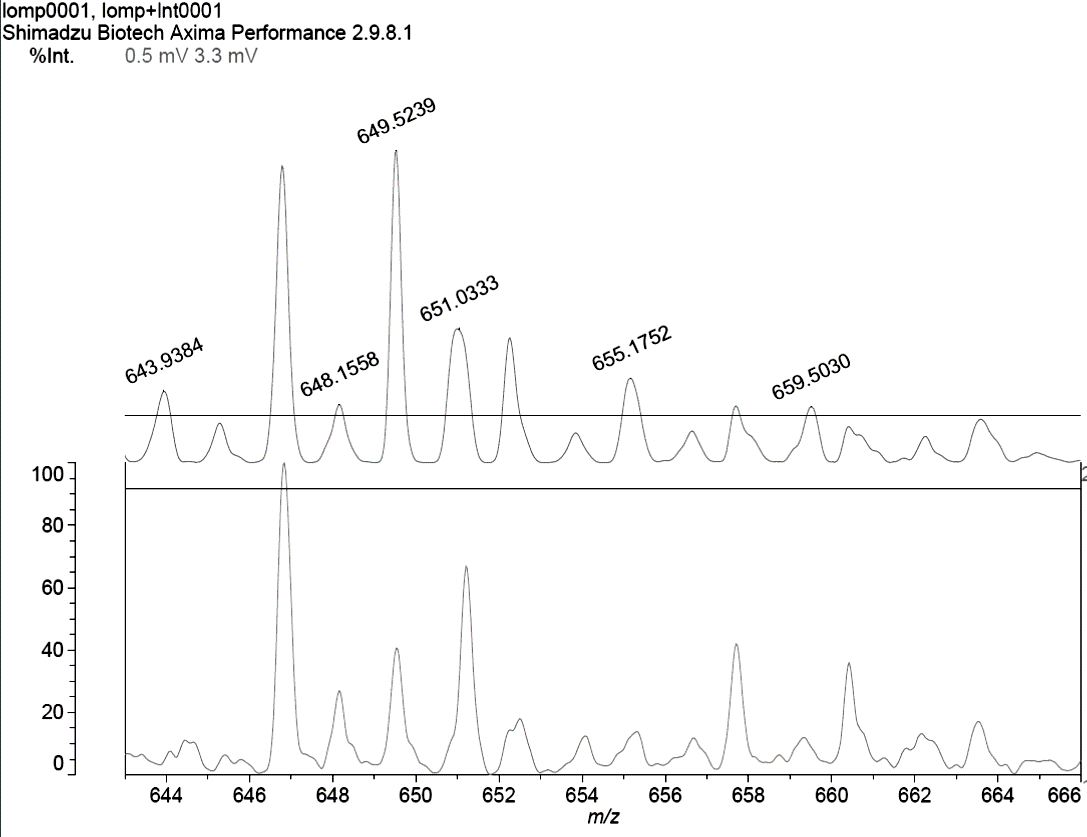


Inset C


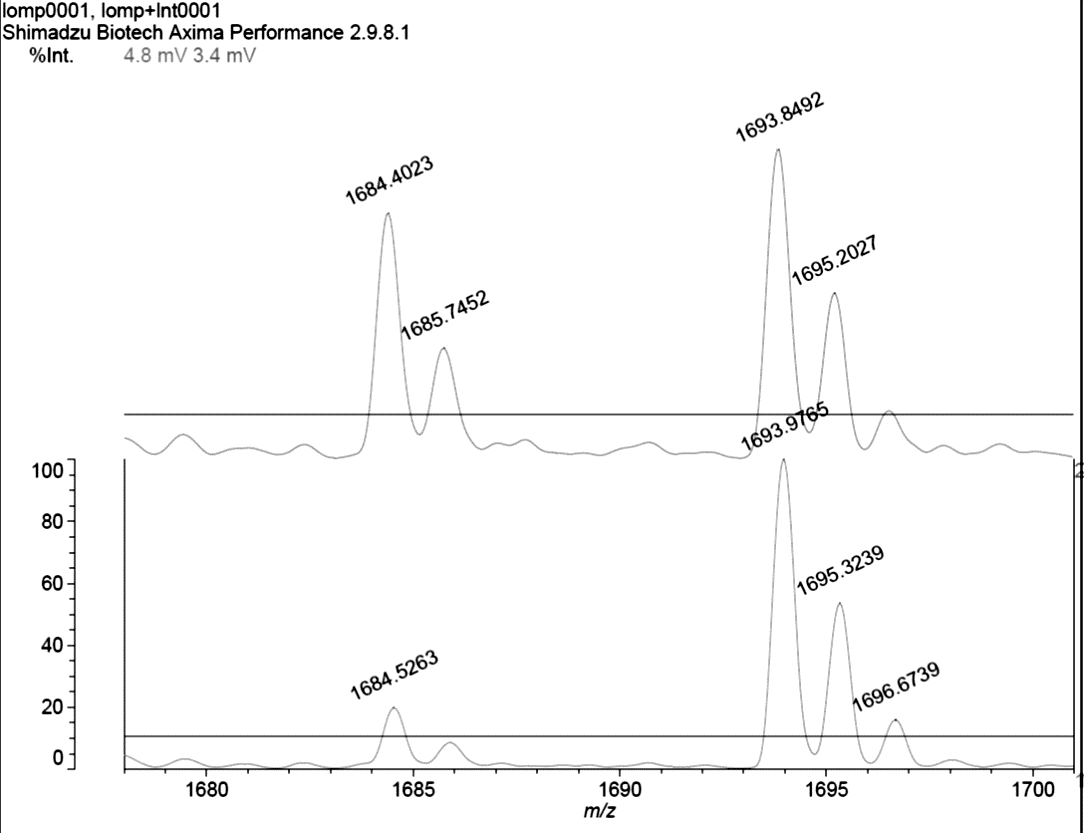


Inset D


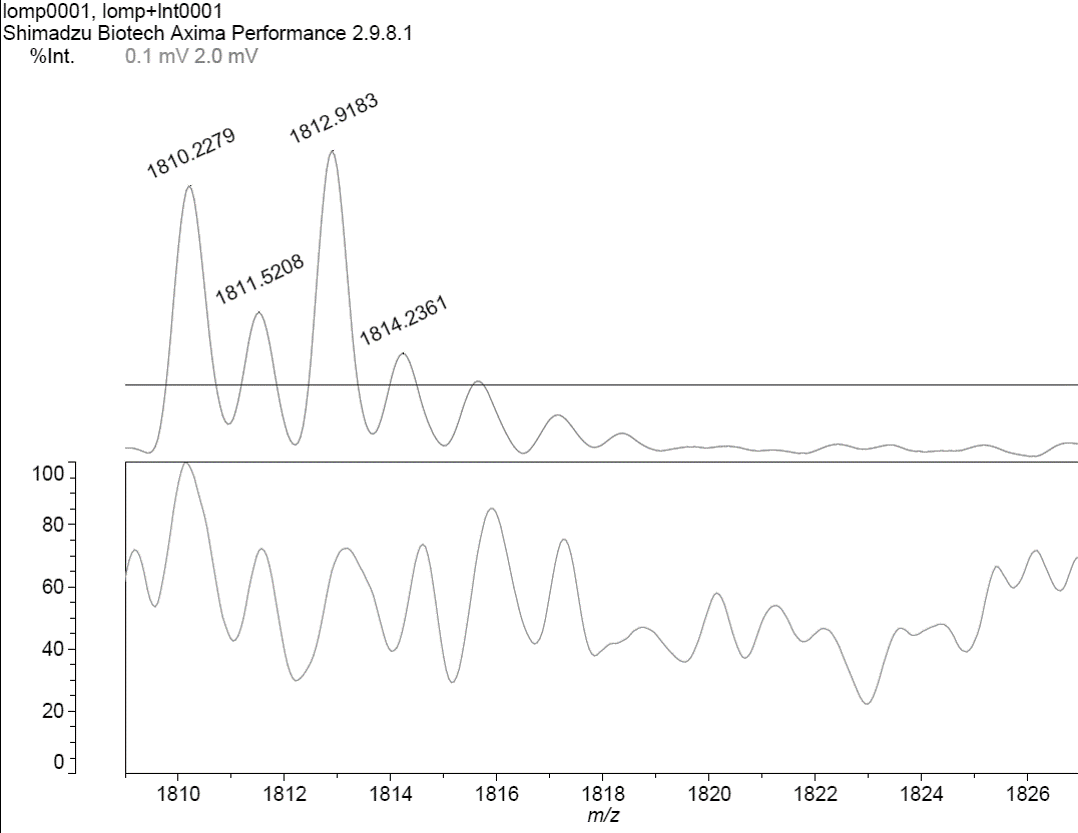

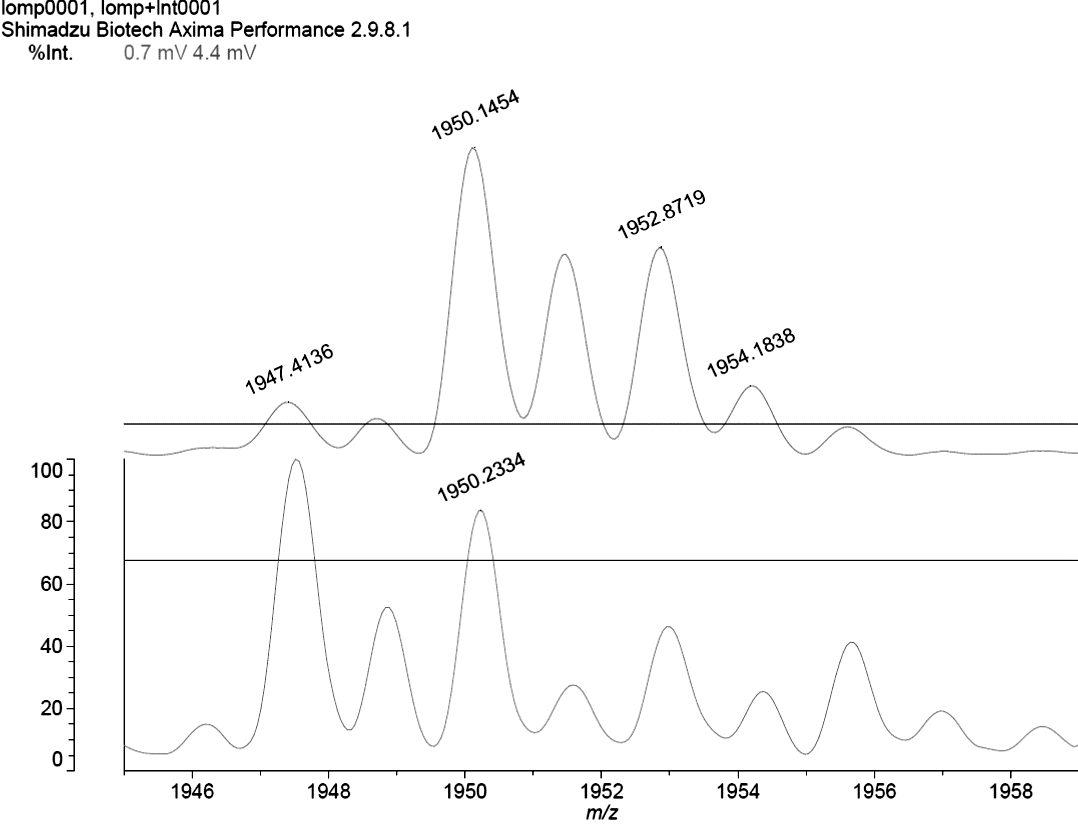

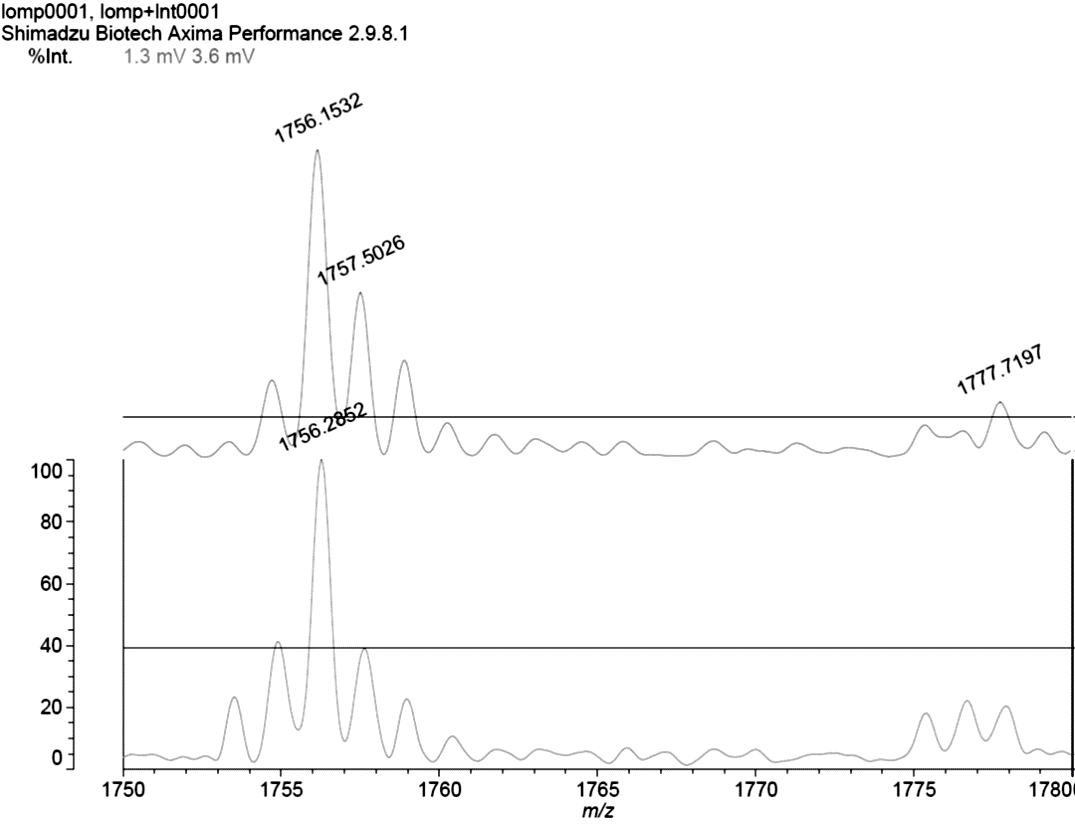


Figure S3: MALDI-TOF experiment of L-OMP26 expressed by *E. coli* in M9-MM media but with and without additional Lnt enzyme. Top with Lnt and bottom without Lnt. Overall same signature in the two spectra. The main differences related to lipidation moiety changes are highlighted (Inset) and shown as zoom of overall spectra (middle top spectra).

**Lipid modification #B’ (Charge +4)**

**Triacyl L-OMP2 Diacyl L-OMP26**

**C16:0, C16:0, C16:1 C16:0, C16:1**


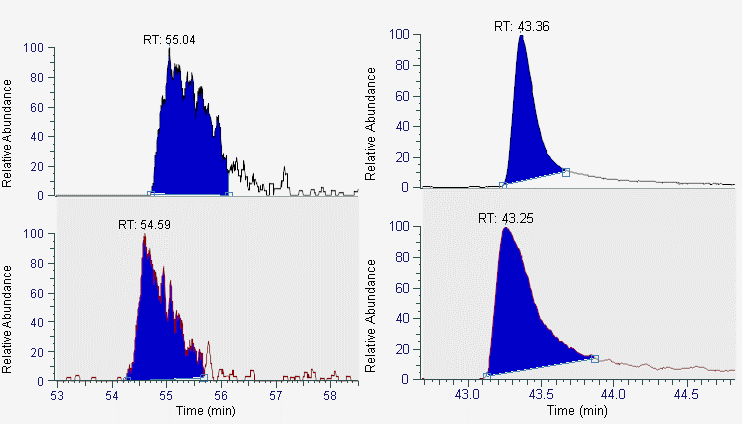


**M9 minimal media**

**+Lnt -Lnt**

Figure S4: Mass spectrometry analysis of L-OMP26 grown in M9-MM without (top) and with Lnt (bottom) showing peak intensities of lipid modifications 1. (A) The peaks represent triacylated (left) and diacylated (right) L-OMP26 peptides. The peak area is highlighted in blue.

Table S2: Fusion MALDI peak comparison

| Triacylated peak (MASS) | Intensity Pam3CSK4  Control/Standard | Intensity L-OMP26φNL-P6  Lnt-M9-MM | Intensity  M9-MM  L-OMP26φNL-P6 | Ratio of peak Intensity  Lnt-M9-MM/M9-MM  L-OMP26φNL-P6 |
| --- | --- | --- | --- | --- |
| 550.3352 | 478 | 4,784 | 966 | 5.0 |
| 568.3153 | 1,639 | 25,720 | 4,092 | 6.3 |
| 569.3350 | Low | 8,463 | 1,300 | 6.5 |
| 578.8558 | 271 | 4,550 | 406 | 11.2 |
| 590.2998 | 168 | 1,480 | 439 | 3.4 |
| 703.5658 | 104 | 1,425 | 487 | 2.9 |
| 704.1163 | 904 | 1,028 | 353 | 2.9 |
| 757.4294 | 109 | 1,514 | 248 | 6.1 |
| 758.5404 | 105 | 631 | 157 | 4 |
| Diacylated peak (MASS) | Intensity Pam2CSK4  Control/Standard | Intensity L-OMP26φNL-P6  Lnt-M9-MM | Intensity  M9-MM  L-OMP26φNL-P6 | Ratio of peak Intensity  Lnt-M9-MM/M9-MM  L-OMP26φNL-P6 |
| 666.205 | 887 | 1348 | 1634 | 0.82 |
| 863.2364 | 182 | 96 | 299 | 0.32 |
| 893.1794 | 578 | 357 | 1027 | 0.35 |
| 1220.5745 | 355 | 173 | 275 | 0.63 |
| 1304.8703 | 2373 | 264 | 253 | 1.04 |
| 1318.6074 | 2412 | 774 | 1335 | 0.58 |
| 1320.6298 | 1734 | 281 | 379 | 0.74 |

Comparison of MALDI spectra peak intensities that were higher (top) or lower (bottom) for L-OMP26φNL-P6 with *E. coli* grown in Lnt-M9-MM (triacylated) than M9-MM

L-OMP26φNL-P6 (diacylated). Pam3CSK4 (triacylated) & Pam2CSK4 (diacylated) were used for comparison as control. Ratio of peak intensity shown indicates Lnt enhanced triacylation.


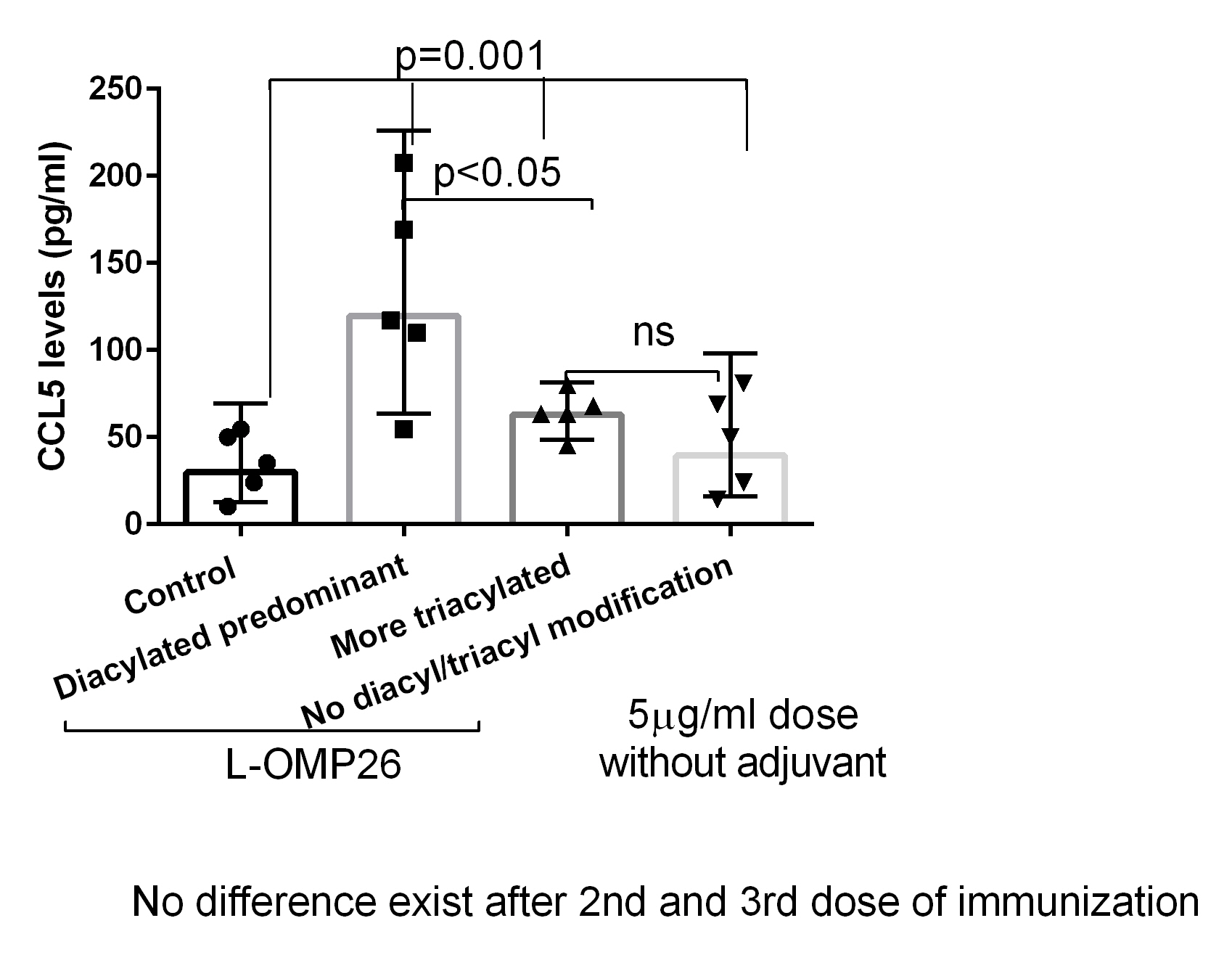


Figure S5: Comparison of CCL5 in mice immunized with different constructs of L-OMP26.

Control is PBS immunization, diacylated predominant L-OMP26 was grown in *E. coli* with M9-MM media, more triacylated L-OMP26 grown in *E. coli* with Lnt-M9-MM and No diacyl/triacyl modification were L-OMP26 grown in 2X-YT. p-values by ANOVA with Tukey multiple comparison. ns=not significant.

Note: Sera was collected 24 hrs. after each L-OMP26 immunization (5µg dose/mice) for reactogenicity.


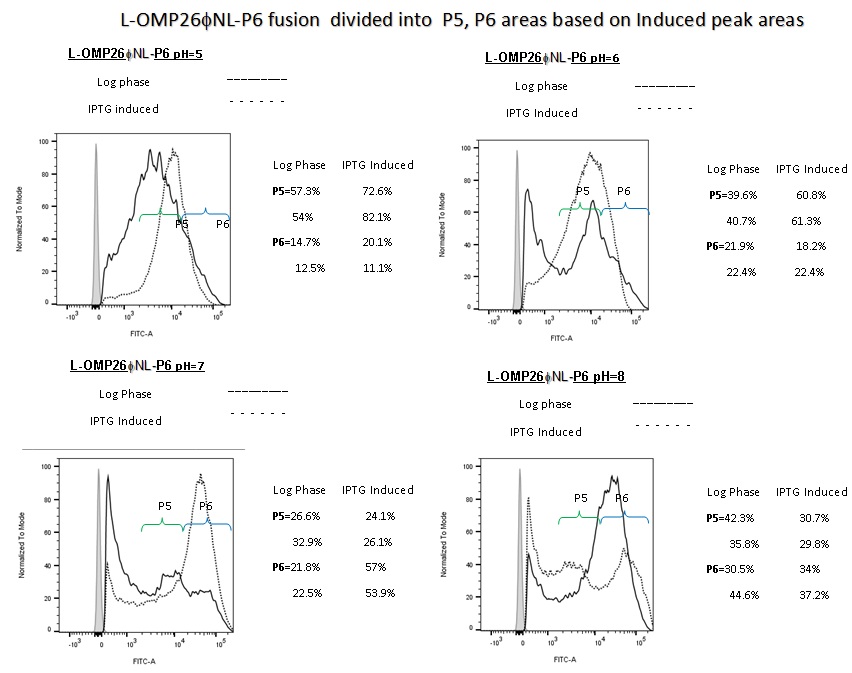


Figure S6: Change in expression of L-OMP26φNL-P6 on the surface of *E. coli* with change in pH at log phase (solid line) and post induction phase (dotted line). Two peaks (P5 and P6) were assigned at log phase. P5 corresponds to diacylated and P6 corresponds to triacylated L-OMP26 in the L-OMP26φNL-P6 fusion construct. Population density area of P5 and P6 peaks shown as proportion expression corresponding to each pH when the *E. coli* were grown to log phase and after IPTG induce, as shown to the right of each sub-figure.


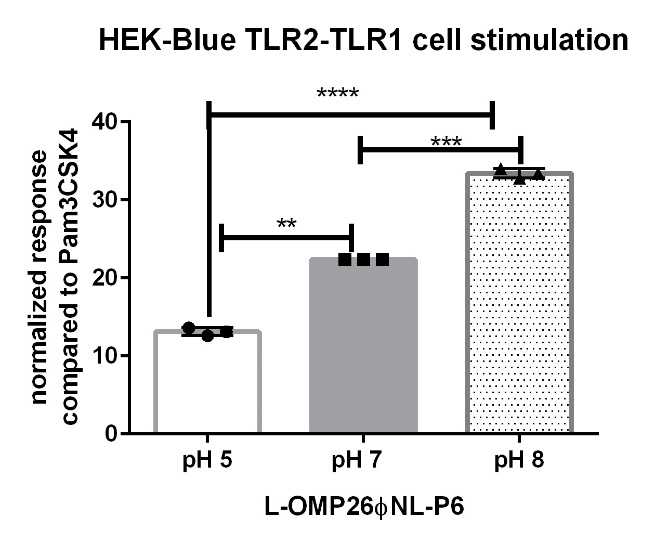


Figure S7: A) HEK-Blue hTLR2-TLR1 cell stimulation by L-OMP26φNL-P6 fusion grown in different pH (5, 7 and 8) conditions. Increased triacyl lipidation occurred with increase in pH. p-value by ANOVA with multiple comparison of normalized OD values of L-OMP26φNL-P6 fusion when *E. coli* were grown in M9-MM with different pH. Y-axis displays OD values normalized against positive control Pam3CSK4.
